# DLK-1/p38 MAP Kinase Signaling Controls Cilium Length by Regulating RAB-5 Mediated Endocytosis in *Caenorhabditis elegans*

**DOI:** 10.1371/journal.pgen.1005733

**Published:** 2015-12-11

**Authors:** Aniek van der Vaart, Suzanne Rademakers, Gert Jansen

**Affiliations:** Department of Cell Biology, Erasmus MC, Rotterdam, the Netherlands; Washington University School of Medicine, UNITED STATES

## Abstract

Cilia are sensory organelles present on almost all vertebrate cells. Cilium length is constant, but varies between cell types, indicating that cilium length is regulated. How this is achieved is unclear, but protein transport in cilia (intraflagellar transport, IFT) plays an important role. Several studies indicate that cilium length and function can be modulated by environmental cues. As a model, we study a *C*. *elegans* mutant that carries a dominant active G protein α subunit (*gpa-3QL*), resulting in altered IFT and short cilia. In a screen for suppressors of the *gpa-3QL* short cilium phenotype, we identified *uev-3*, which encodes an E2 ubiquitin-conjugating enzyme variant that acts in a MAP kinase pathway. Mutation of two other components of this pathway, dual leucine zipper-bearing MAPKKK DLK-1 and p38 MAPK PMK-3, also suppress the *gpa-3QL* short cilium phenotype. However, this suppression seems not to be caused by changes in IFT. The DLK-1/p38 pathway regulates several processes, including microtubule stability and endocytosis. We found that reducing endocytosis by mutating *rabx-5* or *rme-6*, RAB-5 GEFs, or the clathrin heavy chain, suppresses *gpa-3QL*. In addition, *gpa-3QL* animals showed reduced levels of two GFP-tagged proteins involved in endocytosis, RAB-5 and DPY-23, whereas *pmk-3* mutant animals showed accumulation of GFP-tagged RAB-5. Together our results reveal a new role for the DLK-1/p38 MAPK pathway in control of cilium length by regulating RAB-5 mediated endocytosis.

## Author Summary

Cells detect cues in their environment using many different receptor and channel proteins, most of which localize to the plasma membrane of the cell. Some of these receptors and channels localize to a specialized sensory organelle, the primary cilium, that extends from the cell like a small antenna. Almost all cells of the human body have one or more cilia. Defects in cilium structure or function have been implicated in many diseases. Many studies have shown that the length of cilia is regulated and can be modulated by environmental signals. Several genes have been identified that function in cilium length regulation and it is clear that transport of proteins inside the cilium plays an important role. Here, we identify several genes of a MAP kinase cascade that modulate the length of cilia of the nematode *Caenorhabditis elegans*. Interestingly, this regulation seems not to be mediated by the transport system in the cilia, but by modulation of endocytosis. Our results suggest that regulated delivery and removal of proteins and/or lipids at the base of the cilium contributes to the regulation of cilium length.

## Introduction

Primary cilia are evolutionarily conserved organelles that extend from the cell’s surface and are used to sense cues in the environment. Cilia are present on nearly all cells of the vertebrate body and harbor specific receptors and other signaling molecules depending on the cell type. Cilia dysfunction is the cause of many diseases and can result in different symptoms including infertility, polydactyly, retina degeneration, mental retardation and kidney cyst formation [1].

All cilia contain a microtubule core, the axoneme. The axonemal microtubules are essential for a specialized transport pathway called intraflagellar transport (IFT) [2, 3]. IFT transports ciliary building blocks and signaling molecules along the axoneme to the ciliary tip (anterograde) and back to the base (retrograde). Anterograde transport is mediated by kinesin-2 and IFT dynein transports particles in the retrograde direction. In addition to the motors and cargo, IFT particles contain many other proteins, including complex A and complex B and Bardet-Biedl syndrome (BBS) proteins, that are thought to form a scaffold between cargo and motor complexes.

The lipid and protein composition of the cilium differs from that of the plasma membrane to accommodate the cilium’s specialized function [4, 5]. To establish the unique protein and membrane composition, entrance of proteins and lipids is restricted at the base of the cilium by a barrier, called the transition zone [6, 7]. The cilium receives components from multiple sources. One route, originates from the Golgi and involves the Golgi protein GMAP210 and the complex B protein IFT20 [8–10]. In addition, the cilium receives components from endocytic compartments which accumulate at the base of the cilium [11–13]. Disruption of endocytic gene function causes defects in targeting of ciliary transmembrane proteins to the cilium and expansion of ciliary membranes [11, 14, 15]. In mammalian cells, clathrin-dependent endocytosis at the ciliary base is important for the regulation of TGF-β and Notch signaling [16, 17].

Several aspects of endocytosis are regulated by the small GTPase Rab5, including vesicle formation, fusion and motility of early endosomes. Rab5 activity is positively regulated by the guanine nucleotide exchange factor (GEF) Rabex-5 (in *C*. *elegans* RABX-5 and RME-6), while the GTPase activating protein (GAP, TBC-2 in *C*. *elegans*) inactivates Rab5 [18]. In addition, Rab5 membrane localization is regulated by GDP dissociation inhibitors (GDIs). GDI proteins extract the inactive form of most prenylated Rab proteins from membranes and these proteins can subsequently be delivered to target membranes where a new cycle of Rab activation can occur.

We study the structural plasticity of cilia in the nematode *Caenorhabditis elegans*. *C*. *elegans* harbors cilia on the dendritic endings of a subset of neurons, which mainly function in chemosensation. The cilia of the amphid channel neurons are structurally divided in a middle and a distal segment [19, 20]. In these cilia anterograde IFT is mediated by two kinesin-2 motor complexes; heterotrimeric kinesin-II and homodimeric OSM-3 [21, 22]. Imaging experiments have shown that kinesin-II and OSM-3 travel together in the middle segment of the cilium at a velocity of ~0.7 μm/s, while only OSM-3 enters the distal segment where it moves at a higher speed (~1.1 μm/s) [21].

As in other organisms, the structure and function of cilia of *C*. *elegans* are dynamically regulated [23, 24]. Structural changes were observed in cilia of dauer larvae, an alternative larval stage that allows animals to survive for long periods without food [25]. We found a partial uncoupling of the two kinesins in cilia of larvae exposed to a pheromone that induces dauer formation: kinesin-II moved at 0.6 μm/s, while OSM-3 moved at 0.9 μm/s. Complex A and B proteins moved at intermediate speeds [26]. Dauer development involves the ciliary localized heterotrimeric G protein α-subunit GPA-3 [27]. We found that IFT in the cilia of *gpa-3* mutant animals is altered similarly to dauer pheromone exposed animals. In addition, mutants overexpressing a dominant active version of GPA-3 (*gpa-3QL*) have short cilia [26, 27]. It is likely that the uncoupling of the two motor proteins in the *gpa-3QL*(*syIs25*) mutant causes cilia shortening.

To find out how GPA-3 regulates IFT and cilium length we performed a screen for suppressors of the *gpa-3QL* short cilia phenotype. We found that one of the mutants, *sql-4(gj204)* (suppressor of *gpa-3QL* #4), has a mutation in the *uev-3* gene. *uev-3* encodes an E2 ubiquitin-conjugating enzyme variant that binds and regulates the p38 MAP kinase PMK-3 [28]. PMK-3 functions in the conserved DLK-1 and MLK-1 MAP kinase pathways, important for axon development, axon regrowth after injury and synapse formation [28–33]. The DLK-1/p38 MAP kinase pathway is composed of the dual leucine zipper-bearing MAPKKK DLK-1, the MAPKK MKK-4 and the p38 MAPK PMK-3 [29, 31, 32]. The MLK-1/p38 MAPK pathway is composed of the mixed lineage MAPKKK MLK-1, the MAPKK MEK-1 and PMK-3 [32]. Cross-talk between these pathways generates a third pathway, composed of DLK-1, MEK-1 and PMK-3 [32]. p38 MAPK has many downstream targets, such as proteins involved in gene transcription [28, 31], microtubule stabilization [33] and endocytosis [34, 35].

In this study, we show that mutation of several genes in the DLK-1/p38 MAP kinase pathway can suppress the short cilia phenotype of *gpa-3QL* animals. In addition, our results suggest that this pathway acts in cilium length control by regulating RAB-5 mediated endocytosis.

## Results

### Mutation of *uev-3* suppresses the dye-filling defect of *gpa-3QL*

The cilium defects of *gpa-3QL(syIs25*) animals result in diminished uptake of fluorescent dyes in the sensory neurons, a process called dye-filling [20, 27]. To identify new proteins that play a role in this process, we performed a forward genetic screen for suppressors of the *gpa-3QL*(*syIs25*) dye filling defect. Using SNP-mapping we mapped the mutation in *sql-4(gj204)* (suppressor of *gpa-3* *QL* #4) to a region of chromosome I of approximately 280 kb. Sequencing of genes in this region identified a G to A mutation in the first codon of exon 6 of the *uev-3* gene, resulting in a premature stop.

**Fig 1:**
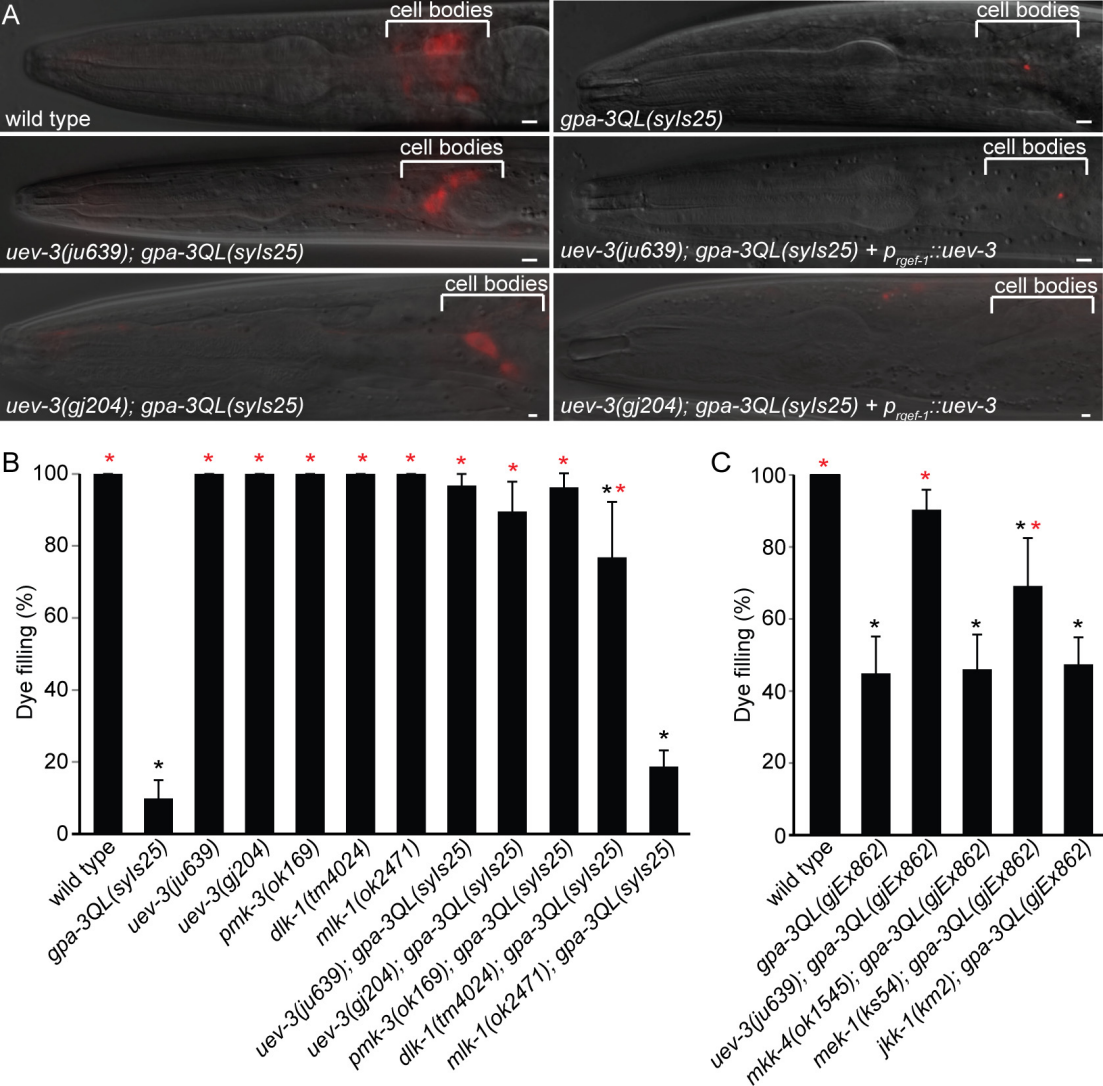
*uev-3*, *dlk-1* and *pmk-3* suppress the *gpa-3QL* dye filling defect. (A) Merge of fluorescence and DIC images of dye-filling of indicated strains. Scale bars 2 μm. Anterior is to the left. (B and C) Percentage dye-filling in the indicated strains. Error bars SD. Statistical analysis was performed using an ANOVA, followed by a Bonferroni post hoc test. Black *: statistically significant compared to wild type, red *: statistically significant compared to *gpa-3QL* (p<0.001).

To verify that this mutation is indeed the suppressor mutation, we analyzed a second *uev-3* allele (*ju639*), which has a 26 base pair deletion in exon 6, resulting in a frame shift and a premature stop. Also *uev-3(ju639)* suppressed the dye-filling defect of *gpa-3QL*(*syIs25*) animals (Fig 1A and 1B). In addition, re-introduction of the wild type *uev-3* gene under control of the pan-neuronal *rgef-1* promoter [28] in *uev-3(gj204); gpa-3QL(syIs25)* or in *uev-3(ju639); gpa-3QL(syIs25)* animals resulted in a dye-filling defect characteristic of *gpa-3QL(syIs25)* animals (Fig 1A). Thus, loss-of-function of *uev-3* suppresses the *gpa-3QL*(*syIs25*) induced dye-filling defect.

### Expression and localization of GPA-3 is not affected by mutation of *uev-3*

Since GPA-3QL expression affects cilium length and dye-filling in a dose-dependent manner [26], we wondered whether the suppressor mutations affect GPA-3QL protein levels and/or localization, thereby restoring dye-filling. Therefore, we performed immuno-fluorescence (IF) using an anti-GPA-3 antibody. GPA-3 is expressed in ten pairs of amphid neurons, in the PHA and PHB phasmid neurons and in the AIZ and PVT interneurons [27, 36]. In wild type animals, GPA-3 is mainly detected in the cilia (S1 Fig). *uev-3(gj204)* and *uev-3(ju639)* animals showed very similar localization of GPA-3 (S1 Fig). In *gpa-3QL*(*syIs25*) animals, GPA-3 localizes to cilia as well as to cell bodies and dendrites [26] (S1 Fig). In the suppressor strains, the localization of GPA-3 and the intensity of the signal was similar to what was observed in the *gpa-3QL*(*syIs25*) mutant (S1 Fig). These results suggest that GPA-3 levels and localization are unaltered in the suppressor strains, although quantitative conclusions cannot be drawn from these experiments.

### Mutation of DLK-1/p38 MAP kinase genes *dlk-1* and *pmk-3* suppress the dye-filling defect of *gpa-3QL*

UEV-3 is an E2 ubiquitin-conjugating enzyme variant shown to directly bind the p38 MAP kinase PMK-3 [28]. To investigate whether PMK-3 is also involved in cilium length control, we tested if mutation of *pmk-3* suppressed the dye filling defect. *pmk-3(ok169); gpa-3QL(syIs25)* animals showed dye-filling, indicating that *pmk-3(ok169)* is also a suppressor of *gpa-3QL(syIs25)* (Fig 1B).

**Fig 2:**
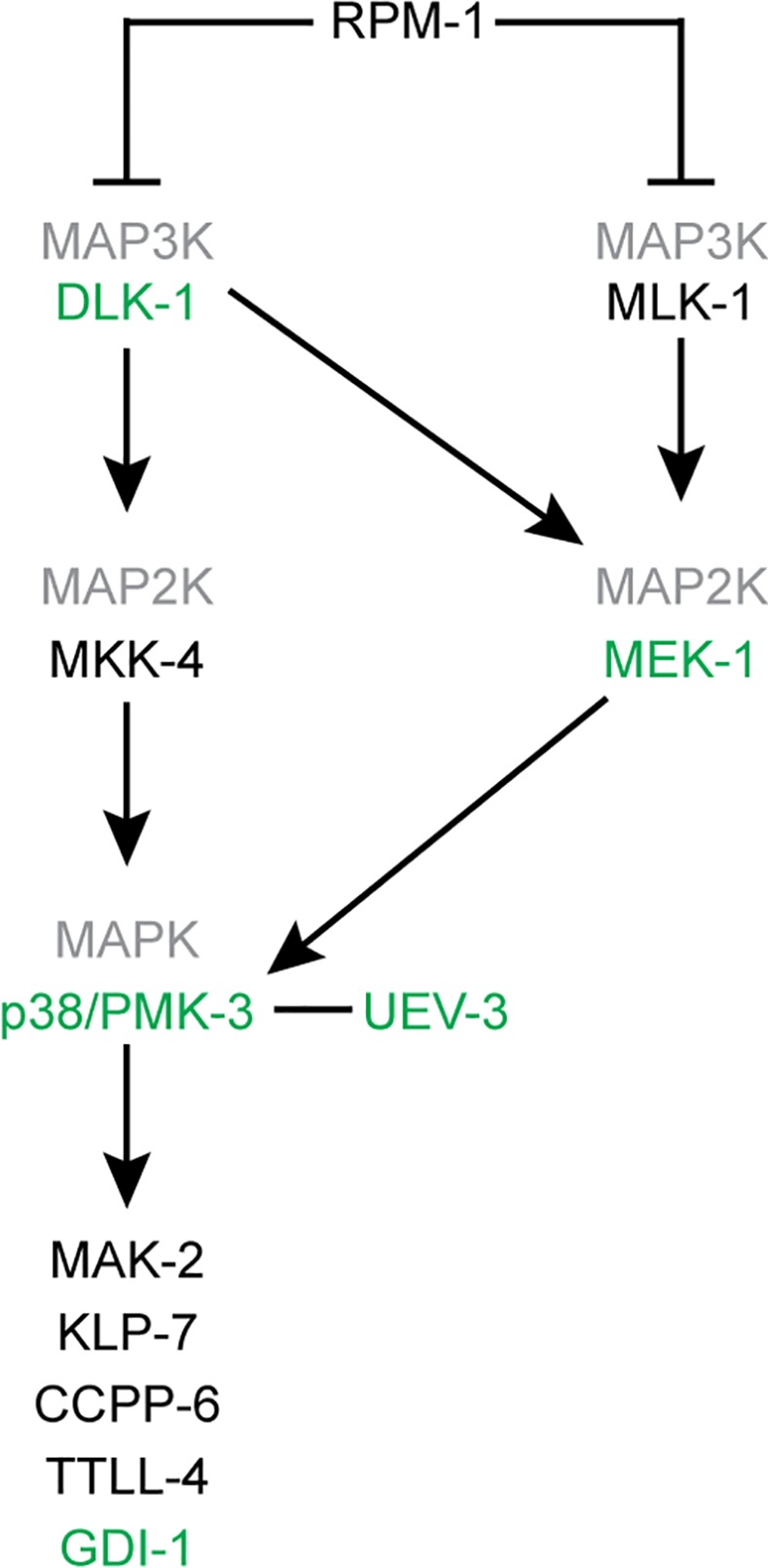
Model of the DLK-1 and MLK-1 MAP kinase pathways. Loss-of-function of proteins indicated in green suppressed the *gpa-3QL* induced dye filling defect. Those in black did not suppress *gpa-3QL*.

PMK-3 functions in the conserved DLK-1 and MLK-1 MAP kinase pathways (Fig 2), important for axon development, axon regrowth after injury and synapse formation [28–32]. The core of the DLK-1 MAP kinase signaling pathway consists of the MAPKKK DLK-1, the MAPKK MKK-4 and the p38 MAPK PMK-3 [28, 29, 31]. The MLK-1 pathway is composed of the MAPKKK MLK-1, the MAPKK MEK-1 and PMK-3 [32]. Cross-talk generates a third pathway composed of DLK-1, MEK-1 and PMK-3 [32].

To investigate whether these pathways are also involved in cilium length control, we tested if loss-of-function of any of these proteins suppressed the dye-filling defect. *dlk-1(tm4024); gpa-3QL*(*syIs25*) animals were dye-filling, whereas *mlk-1(ok2471); gpa-3QL(syIs25*) animals were dye-filling defective, indicating that *dlk-1(tm4024)* is a suppressor of *gpa-3QL*(*syIs25*) (Fig 1B).

Since *mkk-4*, *mek-1* and the *gpa-3QL(syIs25)* transgene reside on the X chromosome, we used an independent extrachromosomal *gpa-3QL* transgene, *gjEx862*, to test if *mkk-4(ok1545)* or *mek-1(ks54)* suppressed the dye-filling defect. First, we established that *uev-3(ju639)* suppressed the dye-filling defect of *gpa-3QL(gjEx862)* animals (Fig 1C). *mek-1(ks54); gpa-3QL(gjEx862*) animals showed a significant increase in dye-filling compared to the *gpa-3QL(gjEx862*) mutant, but did not reach wild type levels (Fig 1C). *mkk-4(ok1545); gpa-3QL(gjEx862)* animals were dye-filling defective (Fig 1C).

Finally, another MAP2K and potential activator of PMK-3 was tested, JKK-1 [37]. Deletion of *jkk-1(km2)* did not suppress the dye-filling defect of *gpa-3QL*(*gjEx862*) animals (Fig 1C).

Our data suggest that MEK-1, possibly acting redundantly with another MAP2K, acts directly upstream of PMK-3 and downstream of DLK-1 in the pathway regulating cilium length in *gpa-3QL* animals (Fig 2).

### Mutation of *dlk-1*, *pmk-3* and *uev-3* restores cilium length of *gpa-3QL* animals cell autonomously

**Fig 3:**
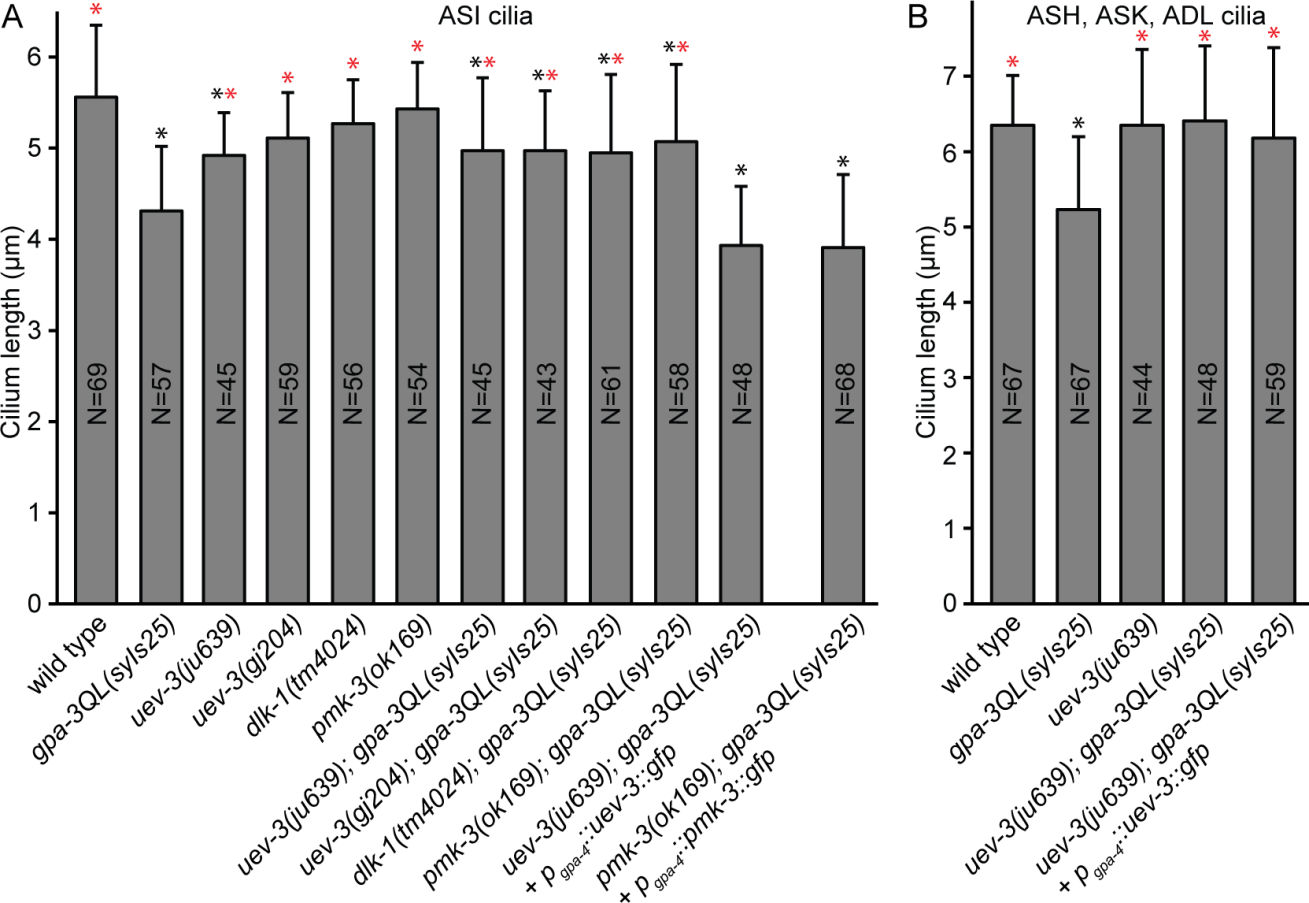
Cilium length is restored in the suppressor mutants. (A) Average lengths of sensory cilia of the ASI neurons in indicated strains. (B) Average lengths of sensory cilia of the ASH, ASK and ADL neurons in indicated strains. Statistical analysis was performed using an ANOVA, followed by a Bonferroni post hoc test. Error bars SD. Black *: statistically significant compared to wild type, red *: statistically significant compared to *gpa-3QL(syIs25)* (for all p≤0.001, except *pmk-3; gpa-3QL* compared to wild type p = 0.006).

Cilium length is reduced in adult *gpa-3QL*(*syIs25*) animals [26]. We tested whether cilium length is restored in the suppressor strains. First, we measured cilium length in the ASI neurons of *uev-3*, *dlk-1* and *pmk-3* single mutants using a *p*_*gpa-4*_::*gfp* construct, resulting in expression of GFP specifically in this pair of neurons. *uev-3(ju639)* animals had slightly shorter cilia than wild type, while the other single mutants showed wild type lengths (Fig 3A). Cilium length in ASI neurons of the suppressor mutants was significantly longer than in *gpa-3QL*(*syIs25*) (Fig 3A). In addition, cilia of the ASH, ASK and ADL cells, visualized using a *p*_*gpa-15*_::*gfp* construct, were restored in the *uev-3(ju639); gpa-3QL*(*syIs25*) mutant (Fig 3B). However, the posterior displacement of the cilia, previously seen in the *gpa-3QL(syIs25)* mutant, was still observed in the suppressor strain (S2 Fig) [26].

To confirm that the effects on cilium length are specific, we expressed *uev-3* or *pmk-3* specifically in the ASI neurons of *uev-3(ju639); gpa-3QL*(*syIs25*) or *pmk-3(ok169)*; *gpa-3QL(syIs25)* mutants, respectively. In both cases, this lead to a decrease in ASI cilium length (Fig 3A). Expression of UEV-3 in the ASI neurons of *uev-3(ju639); gpa-3QL*(*syIs25*) animals did not reduce cilium length in the ASH, ASK and ADL neurons, indicating that the effect of *uev-3* on cilium length is cell autonomous (Fig 3B).

### UEV-3, PMK-3 and DLK-1 localization

We next determined the expression pattern of UEV-3, PMK-3 and DLK-1 in *C*. *elegans*. The *uev-3* gene is part of an operon together with *rab-5*. Trujillo *et al*. have shown that GFP-tagged UEV-3 expressed from a 1.8 kb operon promoter, can be detected in all tissues (S3 Fig) [28].

**Fig 4:**
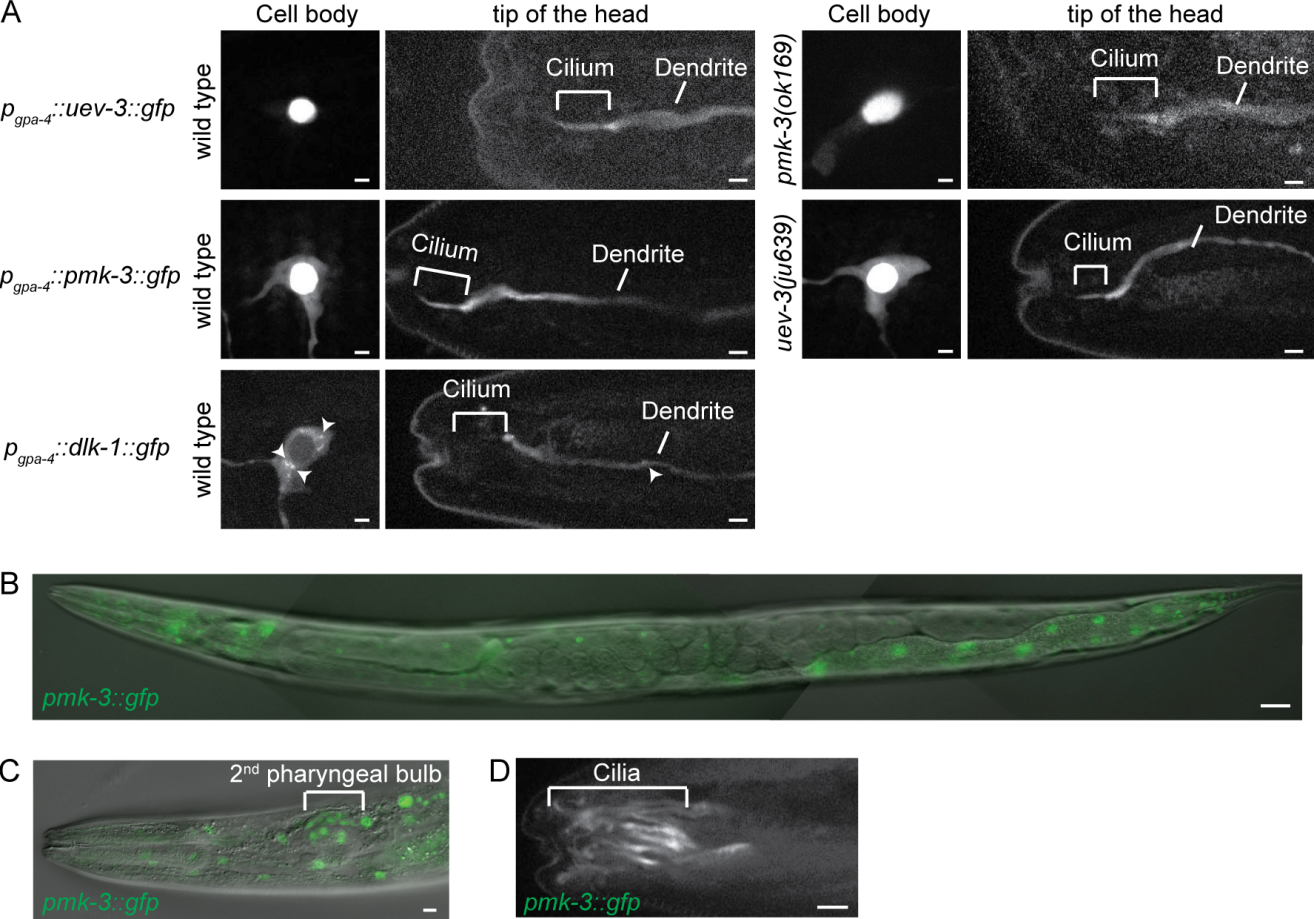
UEV-3, PMK-3 and DLK-1 localization. (A) Fluorescence images of animals expressing either GFP-tagged UEV-3, PMK-3 or DLK-1 in ASI neurons. Arrowheads indicate DLK-1 positive foci. Scale bar 1 μm. (B) Merge of DIC and fluorescence images revealing ubiquitous expression of PMK-3::GFP, expressed from its endogenous promoter. Scale bar 20 μm. (C) Merge of DIC and fluorescence images showing PMK-3::GFP localization in neurons around the 2^nd^ pharyngeal bulb. Scale bar 2 μm. (D) Fluorescence image of PMK-3::GFP localization in cilia of amphid channel neurons. Scale bar 2 μm. Anterior is to the left.

To determine whether UEV-3 localizes to cilia, we expressed UEV-3::GFP from the ASI neuron specific *gpa-4* promoter. This revealed that UEV-3::GFP was predominantly localized to the nuclei of the ASI neurons (Fig 4A), which fits with the presence of a nuclear localization signal (KKRRR) in the C-terminus of the protein. We also detected weak UEV-3::GFP fluorescence in the dendrites, axons and cilia of these neurons (Fig 4A). It would be interesting to determine whether the nuclear localization signal in UEV-3 also plays a role in import into the cilium, as has been found for the kinesin-2 KIF17, where ciliary localization depends on a KRKK sequence and nuclear import proteins [38].

Expression of PMK-3::GFP from its endogenous promoter using a fosmid-based construct confirmed that PMK-3::GFP is widely expressed throughout the animal (Fig 4B) [29, 39, 40]. We observed strong PMK-3::GFP fluorescence in nuclei of neurons surrounding the second pharyngeal bulb, where the cell bodies of the ciliated amphid channel neurons localize (Fig 4C). PMK-3::GFP is mainly present in the nucleus [40], but we also detected PMK-3::GFP in cilia and in the dendrites (Fig 4D). To look more closely at the subcellular localization of PMK-3::GFP, we expressed this fusion protein specifically in ASI neurons. PMK-3::GFP localized mostly to the nucleus of these neurons, but was also visible in the cytoplasm, dendrites, axons and cilia (Fig 4A).

Since UEV-3 directly binds PMK-3 we tested whether the localization of PMK-3::GFP was dependent on UEV-3, or vice versa, by expressing *p*_*gpa-4*_::*pmk-3*::*gfp* in the *uev-3(ju639)* mutant and *p*_*gpa-4*_::*uev-3*::*gfp* in the *pmk-3(ok169)* mutant. No change in localization was detected compared to wild type animals, indicating that the localization of UEV-3::GFP or PMK-3::GFP does not depend on PMK-3 or UEV-3, respectively (Fig 4A).

To determine the localization of DLK-1, we expressed DLK-1::GFP from the ASI specific *gpa-4* promoter. DLK-1::GFP mostly localized to the dendrites and axons of these neurons. The protein accumulated at the base of the cilium, but was only occasionally detected at very low levels inside the cilium (Fig 4A). DLK-1::GFP was present as a diffuse signal and as punctae in the dendrites as well as in the cytoplasm, in accordance with previous observations (Fig 4A) [40]. Some of these punctae were mobile in both the dendrite and cell body (S1 and S2 Videos). As a first step to identify the organelles that harbor these DLK-1::GFP punctae, we performed co-localization experiments combining DLK-1::GFP with mCherry::RAB-5 to visualize endosomes, or with immunofluorescence staining against the Golgi protein SQL-1 [10]. These experiments showed that the DLK-1::GFP punctae are distinct from mCherry::RAB-5 labelled endosomes and from SQL-1 labelled Golgi (S4 Fig). Together these results show that UEV-3::GFP and PMK-3::GFP localize to the nucleus, the cytoplasm, axons, dendrites and cilia and DLK-1::GFP localizes to the cytoplasm, axons and dendrites and at the base of the cilia. Whether the localization inside the cilium and/or at its base is important for their function in cilium length control remains to be determined.

### Intraflagellar transport is altered in *uev-3* and *dlk-1* mutant animals

IFT is affected in *gpa-3QL*(*syIs25*) animals: kinesin-II subunit KAP-1 moves at a lower speed compared to wild type (0.6 μm/s), while OSM-3 has a higher velocity (0.9 μm/s) [26]. We proposed that in *gpa-3QL*(*syIs25*) animals three types of IFT particles exist; particles transported only by kinesin-II (0.5 μm/s), particles transported only by OSM-3 (1.1 μm/s) and particles transported by both motors (0.7 μm/s). Of these, the particles transported by OSM-3 only or by both motors can be transported into the distal segments by OSM-3, whereas particles transported by kinesin-II only will not be able to enter the distal segments, since they do not contain OSM-3. These effects could explain the short cilia observed in *gpa-3QL*(*syIs25*) animals.

We wondered whether mutation of *uev-3*, *pmk-3* or *dlk-1* in *gpa-3QL*(*syIs25*) restores IFT. Surprisingly, the speed of KAP-1::GFP in the amphid channel cilia was reduced to ~0.6 μm/s in the *uev-3(ju639)*, *uev-3(gj402)* and *dlk-1(tm4024)* single mutants (Table 1). Also in the double mutant strains *uev-3(ju639); gpa-3QL*(*syIs25*), *uev-3(gj204); gpa-3QL*(*syIs25*) and *dlk-1(tm4024); gpa-3QL*(*syIs25*), KAP-1::GFP speed was ~0.6 μm/s, similar to that in the *gpa-3QL*(*syIs25*) single mutant [26]. Next, we measured the speed of OSM-3::GFP both in the single *uev-3* and *dlk-1* mutants and in the double mutants. In all the imaged strains OSM-3::GFP speed in the middle segment was increased (ranging from 0.78±0.18 to 0.86±0.19 μm/s), very similar to that measured in *gpa-3QL*(*syIs25*) animals (0.78±0.17 μm/s) (Table 1). These results indicate that the speeds of KAP-1::GFP and OSM-3::GFP are not restored to wild type in the suppressor strains.

**Table 1:** *uev-3* and *dlk-1* mutations do not suppress IFT defects.

		Middle segment			Distal segment		
IFT Protein	Genotype	Average	N	Sign.	Average	N	Sign.
**KAP-1::GFP**	wild type	0.70±0.16	90	^###^	-		
	*gpa-3QL(syIs25)*	0.58±0.11	136	***	-		
	*uev-3(ju369)*	0.59±0.13	164	***	-		
	*uev-3(gj204)*	0.53±0.11	247	***	-		
	*dlk-1(tm4024)*	0.57±0.09	165	***	-		
	*uev-3(ju369);gpa-3QL(syIs25)*	0.59±0.11	275	***	-		
	*uev-3(gj204);gpa-3QL(syIs25)*	0.60±0.13	232	*** ^##^	-		
	*dlk-1(tm4024);gpa-3QL(syIs25)*	0.56±0.10	463	***	-		
**OSM-3::GFP**	wild type	0.73±0.18	316	^#^	1.24±0.25	182	^###^
	*gpa-3QL(syIs25)*	0.78±0.17	267	*	1.13±0.23	178	***
	*uev-3(gj204)*	0.86±0.19	500	*** ^###^	1.38±0.18	190	*** ^###^
	*dlk-1(tm4024)*	0.78±0.18	450	**	1.38±0.23	192	*** ^###^
	*uev-3(gj204);gpa-3QL(syIs25)*	0.80±0.18	470	***	1.10±0.18	173	*** ^##^
	*dlk-1(tm4024);gpa-3QL(syIs25)*	0.81±0.22	451	***	1.14±0.16	178	***
**DAF-10::GFP (complex A)**	*uev-3(ju369)*	0.65±0.16	384		1.11±0.19	285	
	*uev-3(ju369);gpa-3QL(syIs25)*	0.67±0.18	141		1.07±0.20	71	
**CHE-13::GFP (complex B)**	*uev-3(ju369)*	0.63±0.15	440		1.12±0.22	358	
	*uev-3(ju369);gpa-3QL(syIs25)*	0.68±0.21	226		1.05±0.23	144	

Average anterograde IFT velocities (in μm/s ± SD) of KAP-1::GFP, OSM-3::GFP, CHE-13::GFP and DAF-10::GFP in wild type and mutant backgrounds. Statistically significant differences (*** or ^###^ p≤0.001; ** or ^##^ p≤0.005; * or ^#^ p≤0.01) compared to IFT velocities in wild type animals are indicated with an *, compared to those in *gpa-3QL(syIs25)* animals are indicated with a ^#^. Statistical analysis was performed using an ANOVA, followed by a Bonferroni post hoc test. N, number of IFT particles measured.

Interestingly, *dlk-1(tm4024)* and *uev-3(gj204)* single mutants showed increased speeds of OSM-3::GFP in the distal segments of the cilia (Table 1). The functional significance of this finding is not clear, but the increased speed could reflect a change in the composition of the IFT particles, resulting in less drag on the motor proteins and thus a higher speed. More in detail analysis of the composition of the IFT particles and their motility is required to address this issue.

Next, we determined the speeds of other IFT particle components, the complex A subunit DAF-10::GFP (mammalian IFT122) and the complex B subunit CHE-13::GFP (mammalian IFT57). In *gpa-3QL(syIs25)* animals, complex A and B proteins move at a speed intermediate to those of the two kinesins (~0.75 μm/s [26]). In the *uev-3(ju639)* single mutant and in the *uev-3(ju639); gpa-3QL*(*syIs25*) double mutant, DAF-10::GFP and CHE-13::GFP moved at 0.63–0.68 μm/s in the middle segments of the amphid channel cilia (Table 1). Thus, these complex A and B proteins moved at a speed slightly lower than that of wild type and *gpa-3QL(syIs25)* animals.

Taken together, the speeds of the IFT components in the *uev-3* and *dlk-1* single mutants and in the *gpa-3QL* double mutants are very similar to those in the *gpa-3QL* mutants, suggesting that suppression of the *gpa-3QL* induced cilium length defect by mutation of *uev-3* or *dlk-1* is not caused by changes in the IFT machinery.

### Blocking endocytosis suppresses the dye-filling defect of *gpa-3QL*

**Fig 5:**
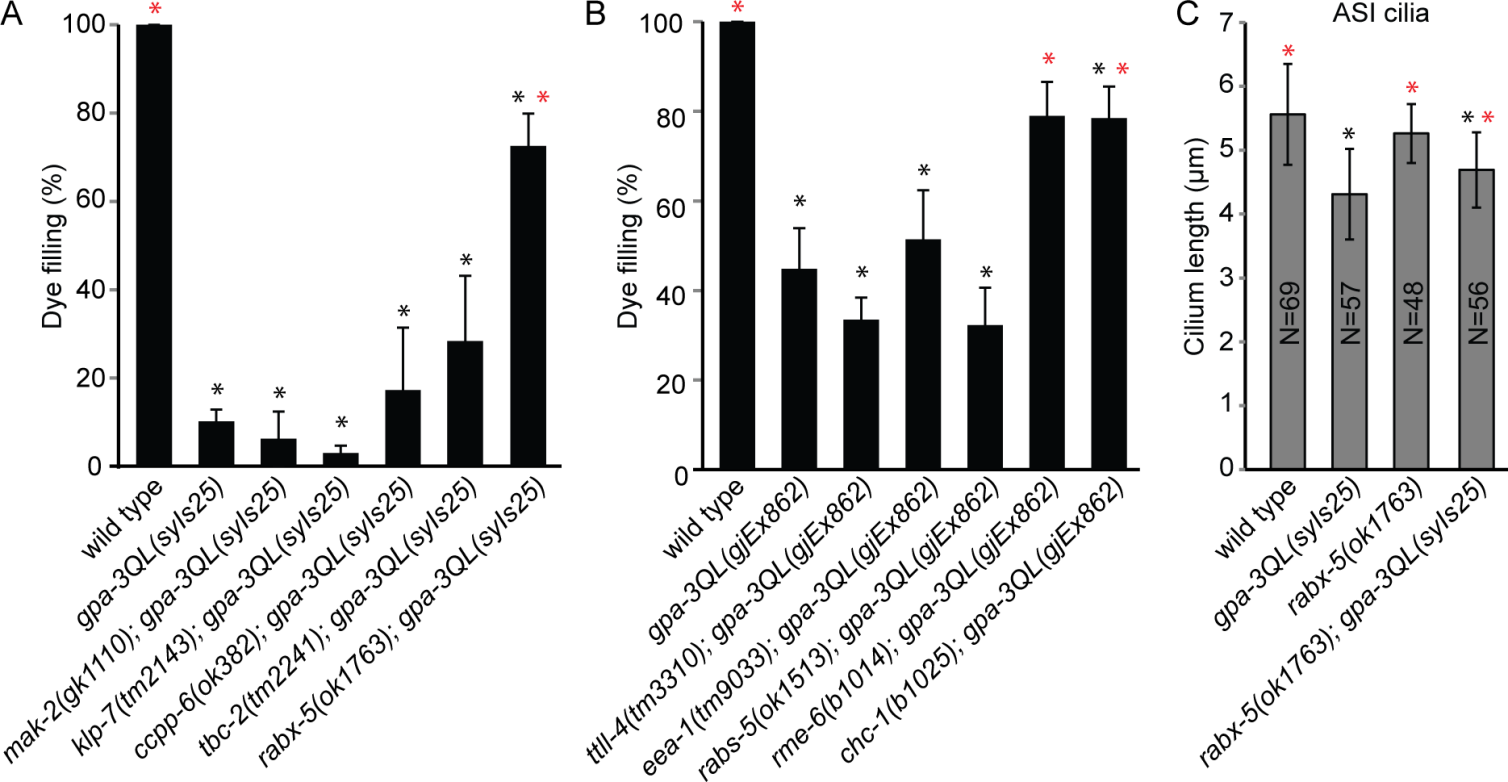
Mutation of the RAB-5 GEFs *rabx-5* and *rme-6* and the clathrin heavy chain *chc-1* suppress the *gpa-3QL* cilium phenotype. (A and B) Percentage dye-filling in the indicated strains. (C) Average length of sensory cilia of the ASI neurons in the indicated strains. Statistical analysis was performed using an ANOVA, followed by a Bonferroni post hoc test. Error bars SD. Black *: statistically significant compared to wild type, red *: statistically significant compared to *gpa-3QL(syIs25)* or *gpa-3QL(gjEx862)* (for all p≤0.001, except in (B) *chc-1; gpa-3QL* compared to wild type p = 0.007, and in (C) *rabx-5; gpa-3QL* compared to *gpa-3QL* p = 0.004).

To identify the mechanism by which PMK-3 regulates cilium length, we tested several downstream effectors of the DLK-1/p38 MAPK pathway. First, we looked at the downstream substrate of PMK-3 in axon regeneration and synapse formation; the MAP kinase-activated protein kinase MAK-2 [28, 31]. *mak-2(gk1110)* did not suppress the dye-filling defect (Fig 5A).

Next, we tested the kinesin-13 family member KLP-7, the cytosolic carboxypeptidase CCPP-6 and the tubulin tyrosine ligase-like TTLL-4 which regulate microtubule dynamics after axon injury [33]. Neither *klp-7(tm2143)*, nor *ccpp-6(ok382)*, or *ttll-4(tm3310)* suppressed the dye-filling defect of *gpa-3QL* (Fig 5A and 5B).

Third, the mammalian homologue of PMK-3, p38 MAPK, was shown to accelerate Rab5-mediated endocytosis by phosphorylating and activating GDI [34, 41, 42]. In addition, p38 MAPK regulates endocytosis by phosphorylating the Rab5 effector proteins EEA1 (EEA-1 in *C*. *elegans*) and Rabenosyn-5 (RABS-5) involved in tethering/docking and fusion of endosomes [35]. Neither *eea-1(tm9033)* or *rabs-5(ok1513)* suppressed the dye-filling defect of *gpa-3QL(gjEx862)* (Fig 5B).

The only GDI homologue in *C*. *elegans*, *gdi-1*, as well as *rab-5* are essential genes, therefore we could not test whether loss of either of these genes suppresses *gpa-3QL*(*syIs25*). Instead, we tested whether mutations in the GEFs *rabx-5* and *rme-6* and the GAP *tbc-2*, which activate and inactivate RAB-5 respectively, are suppressors. Interestingly, *rabx-5(ok1763); gpa-3QL*(*syIs25*) animals are dye-filling, indicating that *rabx-5(ok1763)* is a suppressor of *gpa-3QL*(*syIs25*) (Fig 5A). Cilium length measurements showed that mutation of *rabx-5* significantly restored cilium length in *gpa-3QL(syIs25)* animals (Fig 5C). Also *rme-6(b1014)*, the other RAB-5 GEF, suppressed the *gpa-3QL* induced dye filling defect (Fig 5B). Mutation of the RAB-5 GAP, *tbc-2(tm2241)*, did not suppress the *gpa-3QL* dye filling defect (Fig 5A).

In *C*. *elegans* the p38 MAP kinase pathway was shown to influence AMPA receptor endosomal trafficking at central synapses, indicating that the role of p38 MAP kinase in the regulation of endocytosis is conserved in *C*. *elegans* [40]. This is corroborated by suppression of the *gpa-3QL* cilium defect by mutation of *pmk-3* or the RAB-5 GEFs *rabx-5* and *rme-6*. To establish this further, we tested whether blocking endocytosis by mutating the clathrin heavy chain can also suppress the dye-filling defect. Clathrin is essential for life, so we used a temperature sensitive allele to test its effect on dye-filling. Interestingly, ~80% of *chc-1(b1025); gpa-3QL(gjEx862)* animals cultured at the restrictive temperature of 25°C for 5 days were dye-filling (Fig 5B), indicating that reducing endocytosis by mutating clathrin suppressed the dye-filling defect of *gpa-3QL*. *chc-1(b1025)* single mutants grown at the restrictive temperature were all dye-filling.

### GFP::RAB-5 accumulates in *pmk-3* and *pmk-3; gpa-3QL* animals

**Fig 6:**
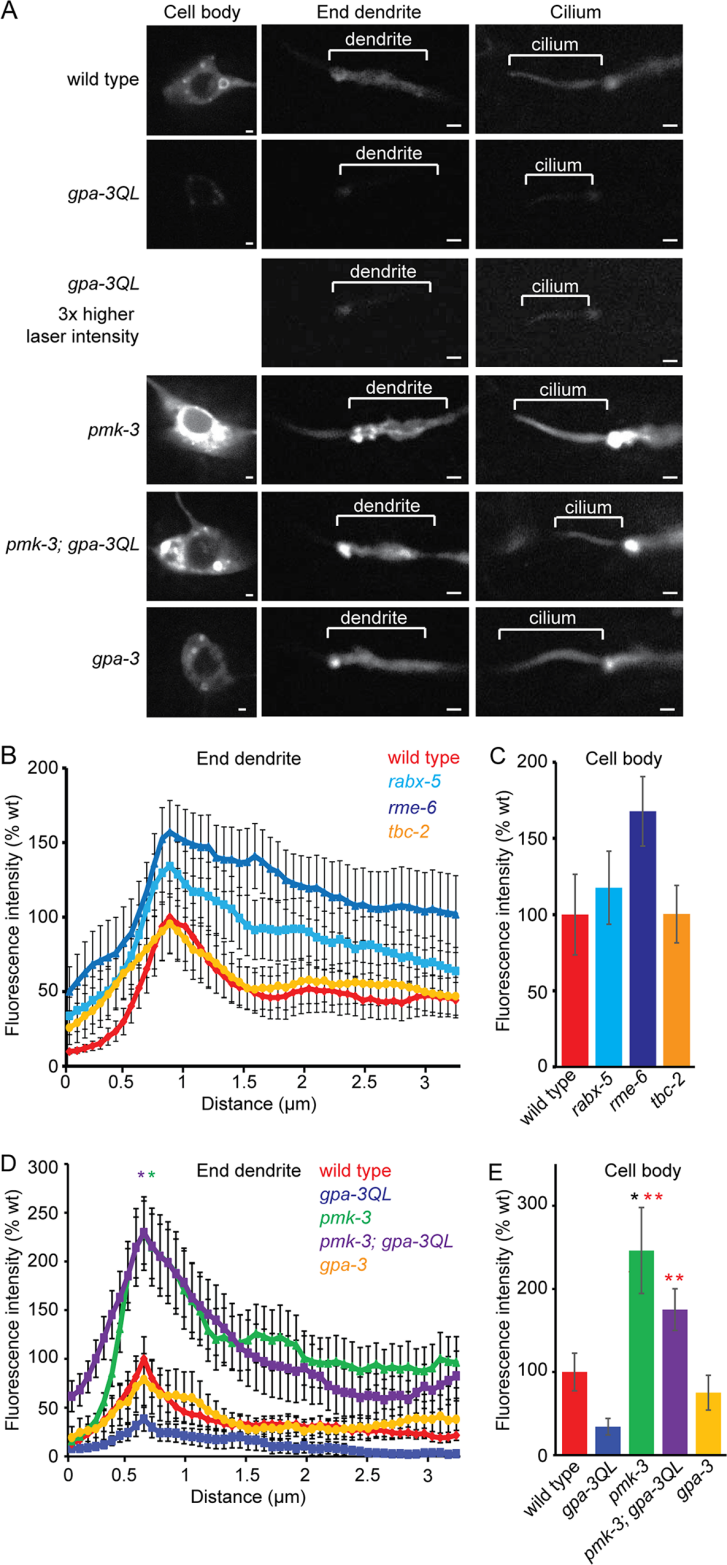
GFP::RAB-5 accumulates in *pmk-3* and *pmk-3; gpa-3QL* mutants. (A) Fluorescence images of GFP::RAB-5 in ASI neurons of indicated strains. Exposure time and laser intensity were kept constant, unless indicated differently. Anterior is to the left. Scale bar 1 μm. (B) Mean fluorescence intensities of dendritic endings of the indicated strains. At least 9 animals were imaged per genotype. No statistically significant differences were observed (p>0.05). (C) Mean fluorescence intensities of ASI cell bodies of the indicated strains, corrected for cell size. At least 9 animals were imaged per genotype. No statistically significant differences were observed (p>0.05). (D) Mean fluorescence intensities of dendritic endings of the indicated strains. 2 independent lines and 16 animals were imaged per genotype. Peaks of *pmk-3(ok169)* (green *) and *pmk-3(ok169); gpa-3QL(syIs25)* (purple *) are significantly different from those of wild type (p<0.05), *gpa-3QL(syIs25)* (p<0.001) and *gpa-3(pk35)* (p<0.005). (E) Mean fluorescence intensities of ASI cell bodies of the indicated strains, corrected for cell size. 2 independent lines and more than 8 animals were imaged per genotype. *pmk-3* animals showed significantly higher fluorescence intensities than wild type (black *; p = 0.005); *pmk-3* and *pmk-3; gpa-3QL* animals showed significantly higher fluorescence intensities than *gpa-3QL* animals (red *; p<0.001). Error bars SEM. Statistical analysis was performed using an ANOVA, followed by a Bonferroni post hoc test.

Our finding that reducing endocytosis can suppress the dye filling defect of *gpa-3QL* animals suggests that increased endocytosis might contribute to the shortening of cilia in these animals. To visualize endocytosis, we expressed GFP::RAB-5 specifically in the ASI neurons. In wild type animals, GFP::RAB-5 localized to the cell body, dendrite and axon. In the cell body, the protein was present in the cytoplasm in punctae and ring structures, presumably early endosomes (Fig 6A). Unlike previous reports, we also detected GFP::RAB-5 inside the cilium (Fig 6A) [11, 15].

First, we analyzed whether GFP::RAB-5 localization was affected by mutation of its GEFs or GAP. Mutation of the RAB-5 GEF *rme-6* resulted in increased GFP::RAB-5 levels, whereas mutation of *rabx-5* only slightly affected GFP::RAB-5 levels, although quantification of the fluorescence intensities did not reveal a significant difference between wild type and *rme-6* or *rabx-5* animals (Fig 6B and 6C). Inactivation of the RAB-5 GAP in *tbc-2(tm2241)* animals did not affect GFP::RAB-5 levels (Fig 6B and 6C). Formal proof that the GFP::RAB-5 construct is functional could not be obtained, because *rab-5* loss-of-function is lethal and this construct is only expressed in the ASI neurons. However, expression of GFP::RAB-5 in the ASI neurons did not affect dye filling, cilium length or speeds of the kinesin-II subunit KAP-1::mCherry or OSM-3::mCherry (S5 Fig).

Next, we tested whether *gpa-3QL* affects GFP::RAB-5. Interestingly, in *gpa-3QL*(*syIs25*) animals GFP::RAB-5 fluorescence intensity was reduced both at the dendritic ending and in the cell body (Fig 6A), although quantification of the fluorescence intensities did not reveal a significant difference between wild type and *gpa-3QL* animals (Fig 6D and 6E). However, these results are in accordance with the hypothesis that endocytosis is affected in *gpa-3QL* animals.

Next, we measured GFP::RAB-5 levels in *pmk-3*(*ok169*), *uev-3(ju639)* and *uev-3(gj204)* animals and in double mutants of these with *gpa-3QL(syIs25)*. In *pmk-3(ok169)* animals, in which presumably GDI-1 is less active, resulting in inactive, membrane bound RAB-5, we would expect accumulation of GFP::RAB-5. Indeed, in these animals GFP::RAB-5 accumulated significantly at the base of the cilium, in the distal part of the dendrite and in the cell body (Fig 6A and 6D and 6E). Similar increases in GFP::RAB-5 fluorescence intensity were observed in the AWB neurons (S6 Fig).

Also *pmk-3*(*ok169*); *gpa-3QL*(*syIs25*) double mutant animals displayed accumulation of GFP::RAB-5 at the ends of the dendrites and in the cell bodies similar to the *pmk-3*(*ok169*) single mutant (Fig 6A and 6D and 6E).

**Fig 7:**
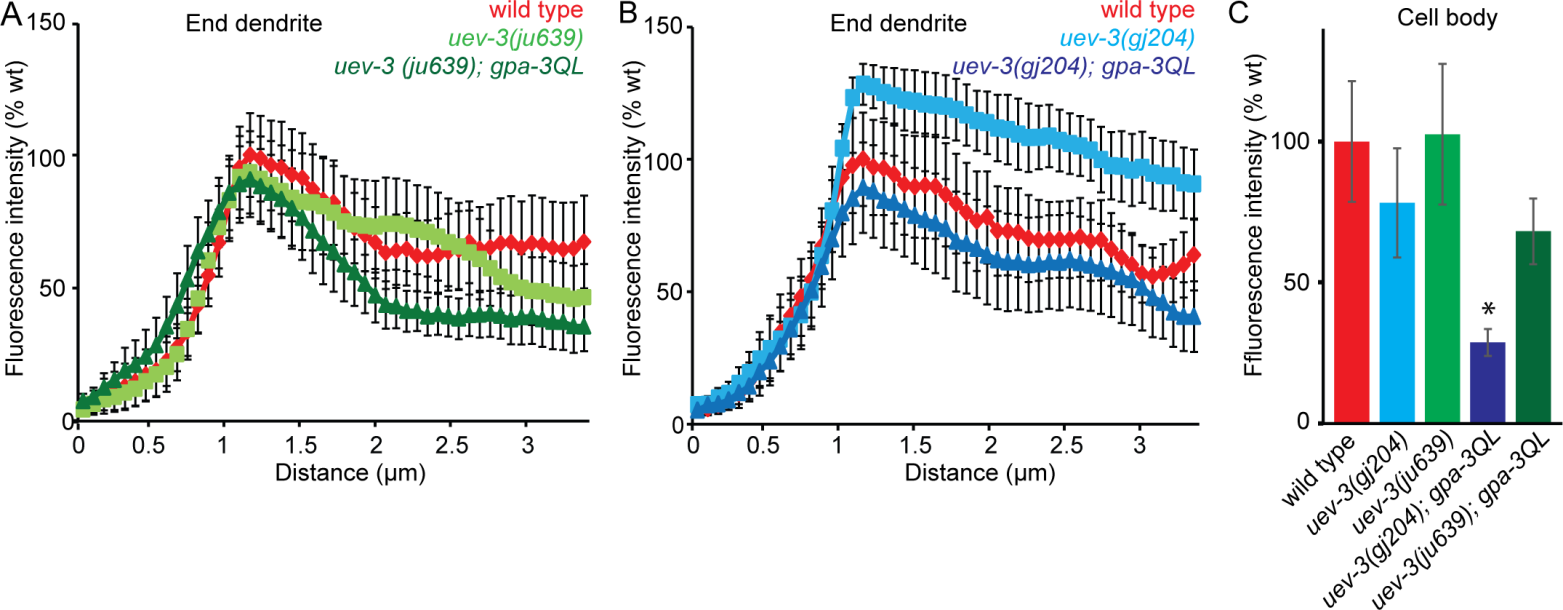
GFP::RAB-5 intensities in *uev-3* and *uev-3; gpa-3QL* animals. (A) Mean fluorescence intensities of dendritic endings of the indicated strains. At least 9 animals were imaged per genotype. No statistically significant differences were observed (p>0.05). (B) Mean fluorescence intensities of dendritic endings of the indicated strains. At least 9 animals were imaged per genotype. No statistically significant differences were observed (p>0.05). (C) Mean fluorescence intensities of ASI cell bodies of the indicated strains, corrected for cell size. At least 9 animals were imaged per genotype. *uev-3(gj204); gpa-3QL* animals showed significantly lower fluorescence intensities than wild type (black *; p<0.05). Error bars SEM. Statistical analysis was performed using an ANOVA, followed by a Bonferroni post hoc test.

GFP::RAB-5 fluorescence intensities in *uev-3(ju639)* and *uev-3(gj204)* mutant animals and in double mutants of both *uev-3* alleles with *gpa-3QL(syIs25)* were not elevated compared to wild type animals (Fig 7A and 7B), suggesting that mutation of *uev-3* has less effect on RAB-5 recycling than mutation of *pmk-3*. However, both *uev-3* mutations restored GFP::RAB-5 levels at the base of the cilium to wild type levels in *gpa-3QL(syIs25)* animals (Fig 7A and 7B), in line with the model that the *dlk-1*/*pmk-3* pathway affects cilium length by reducing RAB-5 mediated endocytosis. Interestingly, in the cell bodies of the ASI neurons, GFP::RAB-5 levels were lower in *uev-3(gj204); gpa-3QL* animals than in wild type animals again suggesting that mutation of *uev-3* has less effect on GFP::RAB-5 recycling than mutation of *pmk-3*. In addition, these results suggest that the *uev-3(gj204)* allele might be weaker than the *uev-3(ju639)* allele, although our other analyses did not reveal such a difference.

Since the animals harboring the dominant active version of GPA-3 showed a slight decrease in fluorescence intensity of GFP::RAB-5, we wondered whether the opposite happens in the *gpa-3(pk35)* loss-of-function mutant. However, *gpa-3(pk35)* animals showed wild type fluorescence intensity levels (Fig 6A and 6D and 6E).

**Fig 8:**
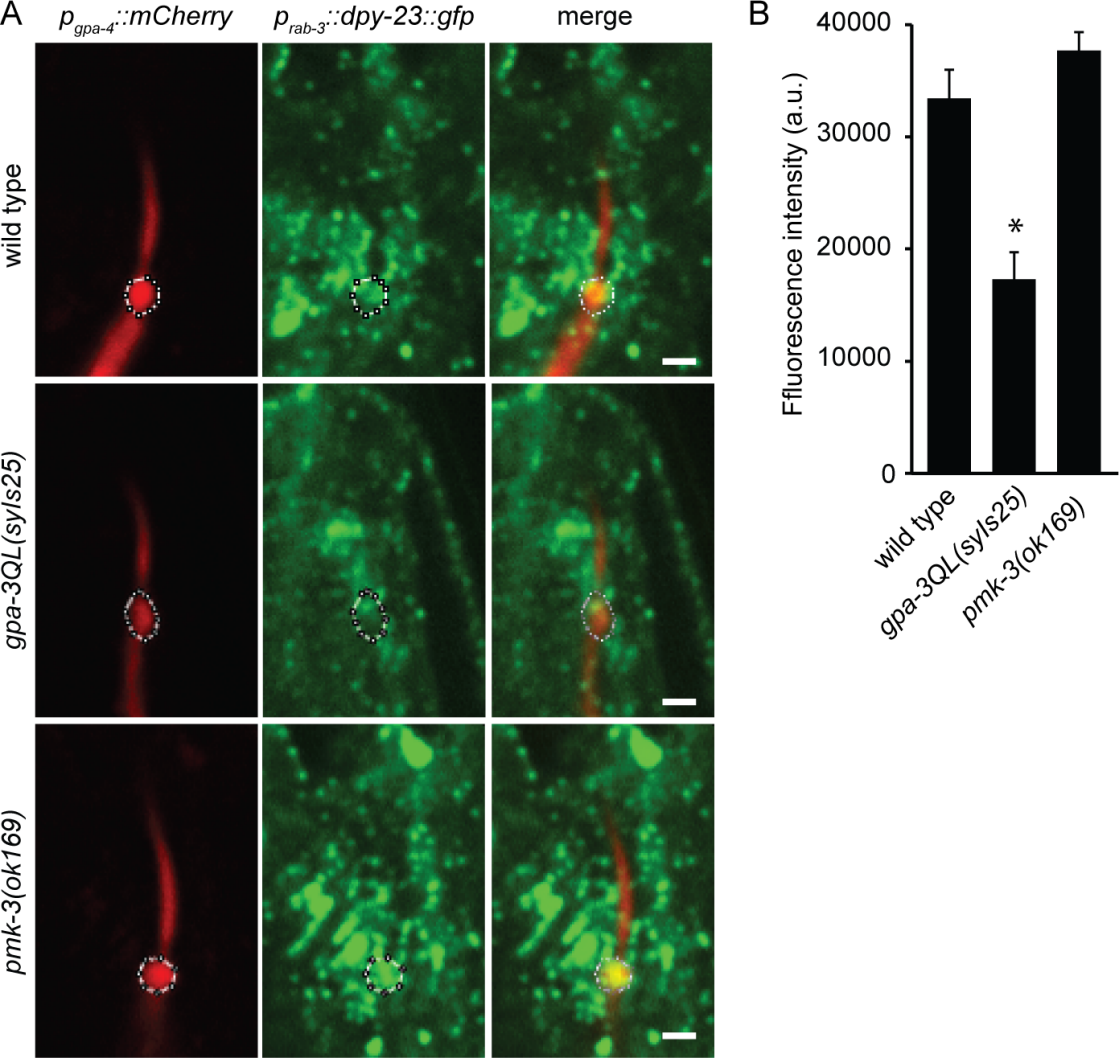
DPY-23::GFP levels are decreased in *gpa-3QL* mutant animals. (A) Fluorescence images of DPY-23::GFP in all neurons, combined with mCherry expressed specifically in the ASI neurons of the indicated strains. The region at the base of the cilium, used to quantify DPY-23::GFP intensity levels, is indicated with a dotted line. Exposure time and laser intensity were kept constant. Anterior is at the top. Scale bar 1 μm. (B) Mean GFP fluorescence intensities of the selected regions at the base of the cilium of the indicated strains. 12 animals were imaged per genotype. Fluorescence intensity in *gpa-3QL(syIs25)* animals was significantly different from wild type (p<0.05). Error bars indicate SEM. Statistical analysis was performed using an ANOVA, followed by a Bonferroni post hoc test.

To confirm that the effects of *gpa-3QL(syIs25)* and *pmk-3(ok169)* on GFP::RAB-5 reflect changes in endocytosis, we visualized DPY-23, the mu2 subunit of adaptor protein complex 2, that mediates endocytosis of membrane proteins. We combined the *p*_*rab-3*_::*dpy-23*::*gfp* construct that expresses the DPY-23::GFP fusion in all neurons [43] with *p*_*gpa-4*_::*mCherry*, to visualize the ASI neurons. Quantification of the DPY-23::GFP fluorescence intensities at the base of the cilia of the ASI neurons revealed significantly lower DPY-23::GFP levels in *gpa-3QL(syIs25)* animals, and wild type levels in *pmk-3(ok169)* animals (Fig 8).

Together, our data show a reduction of GFP::RAB-5 and DPY-23::GFP fluorescence in *gpa-3QL* animals, suggesting an endocytosis defect in these animals. *pmk-3*(*ok169*) and *pmk-3(ok169); gpa-3QL(syIs25)* animals showed accumulation of GFP::RAB-5, but no effect on DPY-23::GFP levels, in line with the expected effect of *pmk-3* mutation on RAB-5 recycling, and probably reflecting reduced endocytosis. Mutation of *uev-3* by itself did not increase GFP::RAB-5 levels, but did restore it to wild type levels in *uev-3; gpa-3QL(syIs25)* animals. Since the accumulated GFP::RAB-5 in *pmk-3* mutant animals probably reflects a reduction of endocytosis, it is likely that *gpa-3QL* causes an increase in endocytosis, which could very well explain the short cilia phenotype of *gpa-3QL* animals.

### GFP::RAB-5 also accumulates in the previously identified suppressor *sql-1; gpa-3QL*

**Fig 9:**
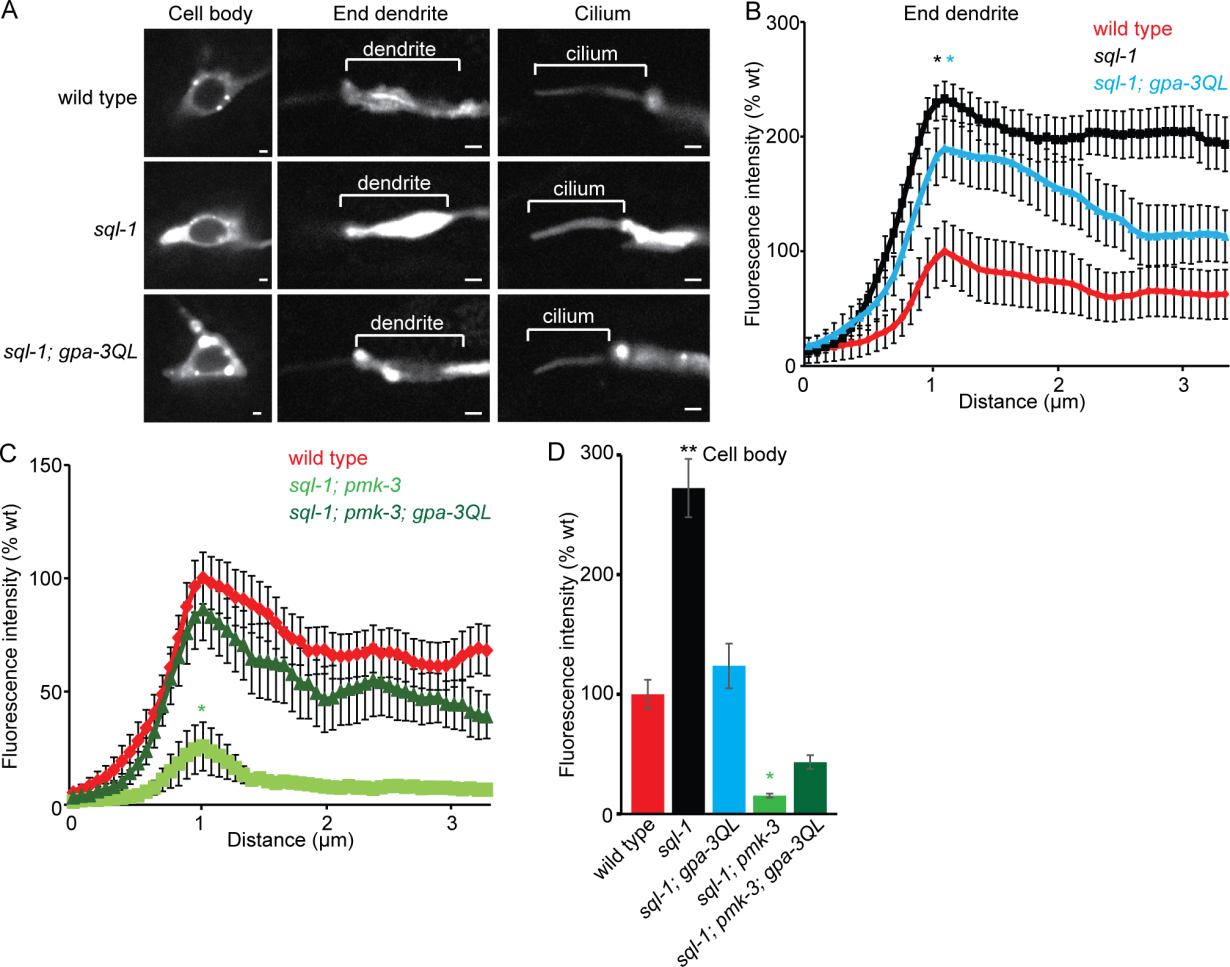
GFP::RAB-5 accumulates in *sql-1* and *sql-1; gpa-3QL* mutants. (A) Fluorescence images of GFP::RAB-5 in ASI neurons of indicated strains. Exposure time and laser intensity were kept constant. Anterior is to the left. Scale bar 1 μm. (B) Mean fluorescence intensities of dendritic endings of the indicated strains. 14 animals were imaged per genotype. Peaks of *sql-1(tm2409)* (black *) and *sql-1(tm2409); gpa-3QL(syIs25)* (blue *) are significantly different from those of wild type (p<0.05). (C) Mean fluorescence intensities of dendritic endings of the indicated strains. At least 13 animals were imaged per genotype. *sql-1; pmk-3* double mutant animals showed significantly lower fluorescence intensities than wild type (p<0.005). (D) Mean fluorescence intensities of ASI cell bodies of the indicated strains, corrected for cell size. At least 7 animals were imaged per genotype. *sql-1(tm2409)* and *sql-1(tm2409); pmk-3(ok169)* animals showed significantly different fluorescence intensities than wild type animals (black *, p<0.001; green *, p<0.05, respectively). Error bars SEM. Statistical analysis was performed using an ANOVA, followed by a Bonferroni post hoc test.

Previously, we reported the identification of another suppressor of the *gpa-3QL* cilium defect, *sql-1* (suppressor of *gpa-3* *QL #1*) [10]. *sql-1* encodes a Golgi protein, the homologue of the mammalian GMAP210, which contributes to maintaining Golgi organization [10, 44]. To test whether mutation of *sql-1* affects endocytosis, we expressed GFP::RAB-5 in the ASI neurons of *sql-1(tm2409)* single and *sql-1(tm2409); gpa-3QL(syIs25)* double mutant animals (Fig 9A). This analysis showed that GFP::RAB-5 accumulated significantly at the base of the cilium of *sql-1* and *sql-1; gpa-3QL*(*syIs25*) animals, and in the cell bodies of *sql-1* animals (Fig 9A and 9B and 9D). These results suggest that both mutation of the *dlk-1/pmk-3* MAP kinase pathway and the Golgi protein *sql-1* affect RAB-5 mediated endocytosis.

To analyze whether *sql-1* and *pmk-3* function in the same genetic pathway in the regulation of GFP::RAB-5 levels we visualized GFP::RAB-5 levels in *sql-1(tm2409); pmk-3(ok169)* double mutant and in *sql-1(tm2409); pmk-3(ok169); gpa-3QL(syIs25)* triple mutant animals. Interestingly, *sql-1(tm2409); pmk-3(ok169)* double mutant animals showed very low GFP::RAB-5 levels, both at the end of the dendrite and in the cell body (Fig 9C and 9D), suggesting that the effects of mutation of *sql-1* and *pmk-3* are mediated by different mechanisms, e.g. by changes in supply of membrane and protein on the one hand and changes in removal on the other. *sql-1(tm2409); pmk-3(ok169); gpa-3QL(syIs25)* triple mutant animals showed similar GFP::RAB-5 levels as wild type animals, consistent with the hypothesis that suppression of the short cilium phenotype of *gpa-3QL* animals by both *sql-1(tm2409)* and *pmk-3(ok169)* involves effects on RAB-5 mediated endocytosis.

## Discussion

We have identified DLK-1/p38 MAP kinase signaling, as a novel pathway that plays a role in the regulation of cilium length. The core of this pathway consists of the dual leucine zipper-bearing MAPKKK DLK-1, which phosphorylates the MAP2K MEK-1 and possibly another redundantly acting kinase, leading to activation of the p38 MAP kinase PMK-3, which binds the E2 ubiquitin-conjugating enzyme variant UEV-3, also required for the function of the pathway. Interestingly, we found that the DLK-1/p38 MAP kinase pathway regulates cilium length by acting on endocytosis. Together our results suggest that in *gpa-3QL* animals endocytosis is enhanced, and that cilium length in these animals can be restored by reducing endocytosis.

How the DLK-1/p38 MAPK pathway is activated is unclear. It could act downstream of GPA-3, since loss-of-function of the DLK-1/p38 MAPK pathway suppresses the dye filling defect of *gpa-3QL*, and loss-of-function of the DLK-1/p38 MAPK pathway and *gpa-3* have similar effects on IFT. However, loss-of-function of *pmk-3* results in accumulation of GFP::RAB-5 at the base of the cilium, whereas loss-of-function of *gpa-3* does not, suggesting that the DLK-1/p38 MAPK pathway might function in parallel to GPA-3, or that the DLK-1/M38 MAPK pathway can be activated by other factors in the absence of GPA-3.

The localization of GFP-fusions of full-length PMK-3, UEV-3 and DLK-1 in or at the base of cilia is consistent with a function in cilia or at their base to directly regulate endocytosis, as also reported by others [34, 40–42]. But since these proteins could also be detected in dendrites and cell bodies and PMK-3 and UEV-3 were most abundant in the nucleus we cannot exclude the possibility that the effects of the DLK-1/p38 MAPK pathway on endocytosis and cilium length are indirect and for example mediated by changes in gene expression. Previous studies have indicated that PMK-3 and its mammalian homologue contribute to stress-induced changes in gene transcription [45, 46].

It is unclear what role UEV-3 plays in the DLK-1/PMK-3 pathway. Although UEV proteins lack catalytic activity, it has been suggested that these proteins have a function in the ubiquitination pathway, probably by forming heterodimers with active E2 ubiquitin-conjugating (UBC) enzymes. For example, UEV-1 interacts with UBC-13 [47, 48] and the mammalian homologues of these proteins catalyze K63 poly-ubiquitin chain formation [49, 50]. K63 ubiquitination has a regulatory role in for example protein localization, protein-protein interaction and transcription. In this way, UEV-3 could regulate PMK-3 activity by regulating its localization. However, we did not observe any effects on PMK-3::GFP localization in *uev-3(ju639)* mutant animals.

Previously, the DLK-1/p38 MAP kinase pathway was shown to be important for axon development, axon regrowth after injury and synapse formation [28–33]. Interestingly, the axon and the cilium share many common features. For example, both are cellular compartments that contain very stable microtubules [33, 51] and both have specialized functions supported by their unique membrane and protein compositions. To maintain these differential compositions, specific proteins and lipids have to be delivered from the cell body. Transport of axonal and ciliary building blocks occurs via microtubule-based transport driven by motor proteins. Recently, it was shown that the DLK-1 pathway acts in axon regrowth after injury by acting on microtubule dynamics via the kinesin-13 family member KLP-7 and the post- translationally modifying enzymes CCPP and TTLL [33]. Although regulation of microtubule stability would nicely fit with regulation of cilium length, mutation of these genes did not suppress the cilium defect of *gpa-3QL(syIs25*) animals.

Instead, we found that the DLK-1/p38 MAP kinase pathway regulates cilium length by acting on endocytosis. Visualization of endosomes using GFP::RAB-5 showed that GFP::RAB-5 levels were lower in the *gpa-3QL* mutant compared to wild type, while they were higher in the *pmk-3*(*ok169)* and the *pmk-3*(*ok169*); *gpa-3QL*(*syIs25*) suppressor mutants. In addition, we showed that the effect of *gpa-3QL* on cilium length can be suppressed by reducing endocytosis, by inactivation of clathrin, or loss-of-function of the RAB-5 GEFs *rme-6* or *rabx-5*. Together these results suggest that in *gpa-3QL* animals endocytosis is enhanced, and that cilium length can be restored by reducing endocytosis.

**Fig 10:**
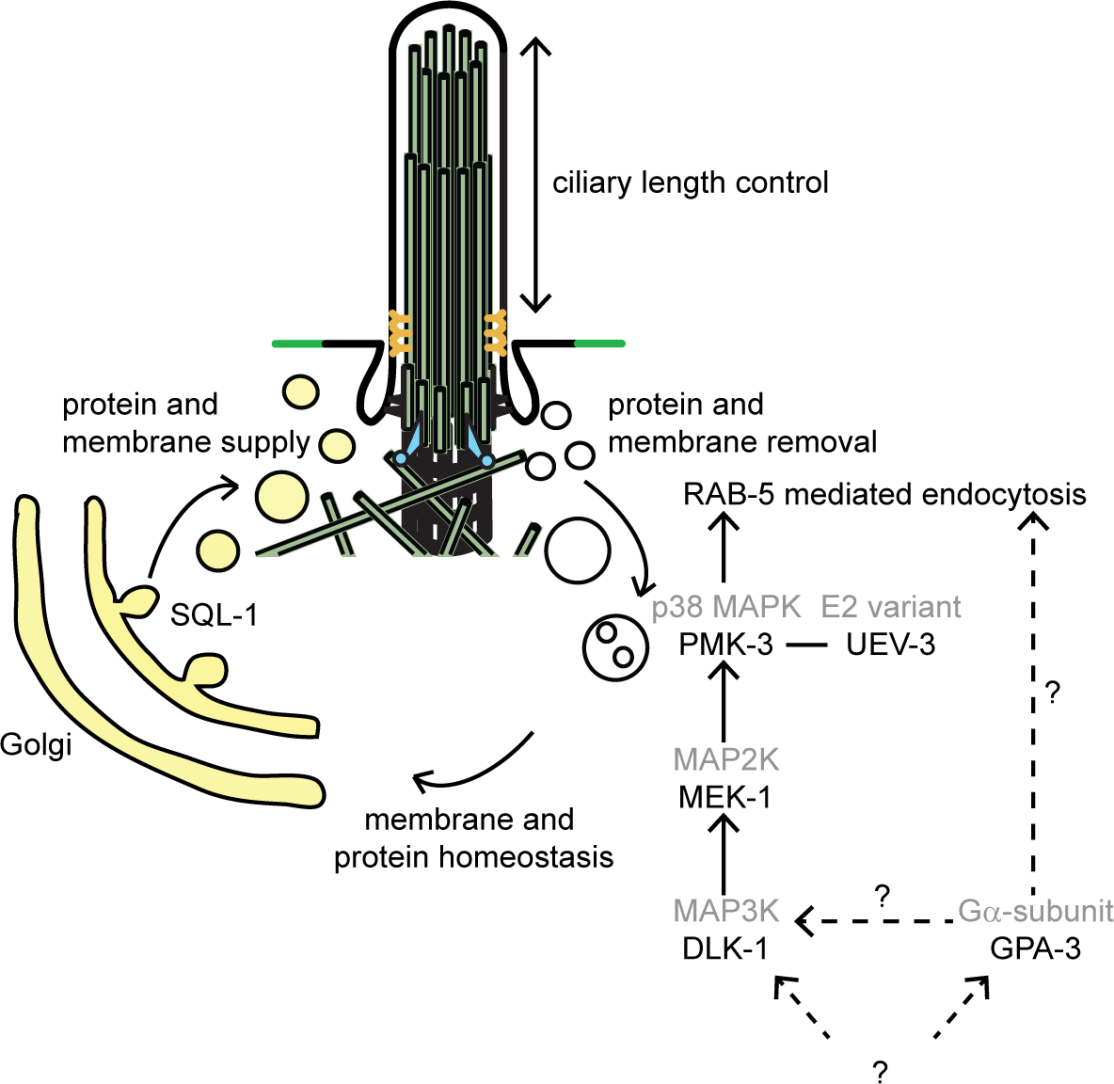
Model of regulation of cilium length by a balance between protein and membrane supply (via the Golgi) and removal (via endocytosis).

How can a change in endocytosis affect cilium length? The removal and supply of ciliary components has to be in balance to maintain cilium length. Removal of ciliary components is probably mediated by active export mediated by the IFT machinery and by endocytosis at the base of the cilium [11, 13, 52]. Thus, increased endocytosis in the *gpa-3QL*(*syIs25*) mutant would result in excessive removal of ciliary membranes and cilia shortening (Fig 10). Reducing endocytosis by reducing RAB-5 activity (by inhibiting DLK-1/p38 MAP kinase signaling and concomitantly GDI activity, or inactivation of the RAB-5 GEFs RME-6 or RABX-5), or by inactivation of clathrin, restores cilium length because the balance of ciliary membrane and protein supply/removal is re-established.

Previously, we identified another suppressor of *gpa-3QL*(*syIs25*), *sql-1* [10]. SQL-1/GMAP210 probably functions in sorting and/or targeting of vesicles from the Golgi to the cilium. To maintain cilium length, both supply and removal of ciliary (membrane) components need to be regulated. Our results suggest that the DLK-1/p38 MAP kinase pathway regulates the removal of ciliary components via endocytosis, while SQL-1 regulates the supply of these components (Fig 10). Indeed, overexpression of SQL-1 leads to an increase in cilium length [10]. In addition, we show that GFP::RAB-5 accumulates in *sql-1* animals, suggesting that endocytosis is affected in these animals. Homeostasis of all membrane enclosed organelles are tightly linked and disruption of one organelle can affect another, for example because membrane dynamics or lipid synthesis is altered [53]. Therefore, it is possible that disruption of the Golgi complex in the *sql-1* mutant has an effect on endocytosis. Our finding that GFP::RAB-5 levels are reduced in *sql-1; pmk-3* mutants, or similar to wild type in *sql-1; pmk-3; gpa-3QL* animals, while they are strongly increased in *sql-1* or *pmk-3* single mutants, are consistent with the hypothesis that SQL-1 and PMK-3 affect GFP::RAB-5 levels via different mechanisms. The clathrin heavy chain, a subunit of the clathrin coat, localizes at the plasma membrane, the Golgi complex and the endosomal compartments where it functions in the formation of transport vesicles [53]. Thus, the suppression of the *gpa-3QL* dye-filling defect by inhibition of *chc-1* could be caused by decreasing endosome formation, but also by altering the formation of clathrin-coated transport vesicles from the Golgi.

Based on our previous work, we proposed that dauer pheromone in the environment of the animal, detected by receptors in the cilia that activate the heterotrimeric G protein α subunit GPA-3, can modulate cilium length by changing the coordination of the two kinesins that mediate anterograde transport in the cilia, resulting in reduced transport of ciliary proteins into the distal segments. Our results presented here suggest that in addition in *gpa-3QL* animals the balance of supply (via the Golgi) and removal (via endocytosis) of membrane and protein components of the cilia is disturbed, resulting in shorter cilia (Fig 10). It will be interesting to determine if similar mechanisms contribute to structural plasticity of cilia in other organisms, allowing the regulation of sensory capacity in response to environmental signals.

## Materials and Methods

### Strains and constructs

Animals were cultured using standard methods [54]. The alleles used in this study were Bristol N2 (wild type), *ccpp-6(ok382)II*, *chc-1(b1025)II*, *dlk-1(tm4024)I*, *eea-1(tm9033)V*, *gpa-3QL(syIs25)X*, *gpa-3QL(gjEx862)*, *gpa-3(pk35)V*, *jkk-1(km2)X*, *klp-7(tm2143)*, *mak-2(gk1110)IV*, *mek-1(ks54)X*, *mkk-4(ok1545)X*, *mlk-1(2471)V*, *pmk-3(ok169)IV*, *rabs-5(ok1513)*, *rabx-5(ok1763)III*, *rme-6(b1014)X*, *sql-1(tm2409)III*, *tbc-2(tm2241)II*, *ttll-4(tm3310)III*, *uev-3(ju639)I* and *uev-3(gj204)I*.

Published transgenes used were: *p*_*gpa-4*_::*gfp* (pGJ325), *p*_*gpa-15*_::*gfp* [55], *kap-1*::*gfp*, *osm-3*::*gfp* and *daf-10*::*gfp* [10], *che-13*::*gfp* [56], *p*_*str-1*_::*gfp*::*rab-5* [11], *p*_*operon-rab-5-uev-3*_::*gfp* (pOF163), *p*_*rgef*_::*uev-3* (pOF112) [28] and *p*_*rab-3*_::*dpy-23*::*gfp* [43]. The fosmid (clone 2666739528585155 H05) containing the complete *pmk-3* gene fused with GFP was obtained from the TransgeneOme Unit of the MPI Dresden [39].

*p*_*gpa-4*_::*uev-3*::*gfp*, *p*_*gpa-4*_::*pmk-3*::*gfp* and *p*_*gpa-4*_::*dlk-1*::*gfp* were created by subcloning the cDNA of *uev-3*, *pmk-3* or *dlk-1* into pGJ325 in frame with *gfp*. *p*_*gpa-4*_::*gfp*::*rab-5* was created by cloning *gfp*::*rab-5* (1.2 kb), obtained by PCR performed on animals expressing *p*_*str-1*_::*gfp*::*rab-5* [11], in a pPD95.77 vector (gift from Andrew Fire) and subsequently subcloned in pGJ325. *p*_*gpa-4*_::*mCherry*::*rab-5* was generated by swapping *gfp* in the *gfp*::*rab-5* construct for *mCherry* (gBlock gene fragment ordered with IDT) and cloning the resulting *mCherry*::*rab-5* fragment into pGJ325. *p*_*gpa-4*_::*mCherry* was created by swapping *gfp* for *mCherry* subcloned from pRSET-B mCherry (gift from Roger Tsien) into pGJ325. *p*_*gpa-4*_::*kap-1*::*mCherry* and *p*_*gpa-4*_::*osm-3*::*mCherry* were created by subcloning *kap-1* or *osm-3* cDNA fragments obtained from *p*_*sra-6*_::*kap-1*::*gfp* or *p*_*sra-6*_::*osm-3*::*gfp* [57] (gift from Piali Sengupta) behind the *gpa-4* promoter, in frame with *mCherry* in the *p*_*gpa-4*_::*mCherry* construct.

Microinjections were performed as described [58]. IFT constructs were injected at concentrations ranging from 15 ng to 75 ng/μl and analyzed for correct expression by imaging IFT particles in wild type animals. Other constructs were injected at concentrations ranging from 10–75 ng/μl.

### Identification and characterization of *sql-4(gj204)*

*gpa-3QL(syIs25)X* animals were mutagenized with 50 mM EMS to generate random mutations. 100 cultures were started with 12 EMS-treated animals each. The F2 and F3 progeny of the mutagenized animals were subjected to dye-filling and 12 animals that took up fluorescent dye were individually picked onto new culture dishes. The progeny of these putative suppressor mutants was subjected to dye-filling. This identified nine independent suppressor mutants, including *sql-4(gj204)*. Using SNP mapping [59], we mapped *sql-4(gj204)* to a region of 280 kb of chromosome I. Sequencing genes in this region identified an G to A mutation in the 6^th^ codon of *uev-3*.

### Dye-filling, immunofluorescence and microscopy

Dye-filling was performed using 0.1 mg/ml DiI (Molecular Probes) as described previously [20]. Each mutant was dye-filled in at least 3 independent experiments and at least 45 adults were counted per experiment. The temperature sensitive *chc-1(b1025)II* strain was grown at the restrictive temperature of 25°C for 5 days before being dye-filled.

For immunofluorescence, animals were permeabilized, fixed and stained according to standard methods [60]. GPA-3 and SQL-1 antibody staining was performed as described [10, 36].

Dye-filling, antibody staining, *p*_*gpa-4*_::*gfp*, *p*_*gpa-15*_::*gfp* and fosmid expressed *pmk-3*::*gfp* fluorescence was imaged at RT using a Zeiss Imager Z1 microscope, 40x (n.a. 1.0), 63x (n.a. 1.4) or 100x (n.a. 1.4) oil immersion objectives, AxioCam NRm camera and Zeiss ZEN software.

Localization of GFP::RAB-5, mCherry::RAB-5, DPY-23::GFP, UEV-3::GFP, PMK-3::GFP and DLK-1::GFP was imaged at RT using a Nikon Ti Eclipse microscope with Spinning Disc unit (CSU-X1, Yokogawa), 100x Plan APO TIRF objective (n.a. 1.49), Photometrics QuantEM:512C EM CCD camera and Metamorph imaging software. During GFP::RAB-5 and DPY-23::GFP imaging, laser intensity and exposure time were kept constant.

Quantification of intensity measurements was done using ImageJ.

### Live imaging intraflagellar transport

Live imaging of the GFP-tagged IFT motor proteins was performed as described [10]. Animals were mounted on a 2% agarose pad and anaesthetized with 10 mM levamisole. Images were acquired imaged at RT using a Nikon Ti Eclipse microscope with Spinning Disc unit (CSU-X1, Yokogawa), 100x Plan APO TIRF objective (n.a. 1.49), Photometrics QuantEM:512C EM CCD camera and Metamorph imaging software. Exposure time 300 ms, 150 frames. Kymographs were generated using ImageJ with the kymograph plugin written by Dr. I. Smal (Erasmus MC).

## Supporting Information

S1 Fig Mutation of *uev-3* does not affect GPA-3QL expression levels or location. Merge of fluorescence and DIC images of wild type, *uev-3(gj204)* and *uev-3(ju639)* animals and two images of *gpa-3QL(syIs25)*, *uev-3(ju639); gpa-3QL(syIs25)* and *uev-3(gj204); gpa-3QL(syIs25)* animals stained with anti-GPA-3 antibodies. GPA-3 localizes to the cilia in wild type, *uev-3(gj204)* and *uev-3(ju639)* animals. GPA-3 levels are strongly increased in *gpa-3QL* animals, resulting in anti-GPA-3 staining in dendrites and cell bodies. This anti-GPA-3 staining is not affected by mutation of *uev-3*. Anterior is towards the left. Exposure time was kept constant.
(TIF)

S2 Fig Posterior displacement of cilia is not affected by mutation of *uev-3*. Fluorescence images of GFP in cilia of ASH, ASK and ADL neurons of wild type and *uev-3(ju639); gpa-3QL(syIs25)* worms using *p*_*gpa-15*_::*gfp*. In *gpa-3QL* animals cilia are often posteriorly displaced (Burghoorn et al, 2010). Similar displacement was observed in *uev-3(ju639); gpa-3QL(syIs25)* worms (arrowhead). Scale bar 2 μm.
(TIF)

S3 Fig *uev-3* is ubiquitously expressed. Fluorescence image of adult worm expressing UEV-3::GFP from the 1.8-kb promoter of the *rab-5-uev-3* operon, schematically depicted above the image (pOF163; Trujillo et al, 2010).
(TIF)

S4 Fig DLK-1::GFP does not colocalize with GFP::RAB-5 labeled endosomes or SQL-1 labeled Golgi. (A) Fluorescence images of DLK-1::GFP (using *p*_*gpa-4*_::*dlk-1*::*gfp*) and mCherry::RAB-5 (using *p*_*gpa-4*_::*mCherry*::*rab-5)* in the cell body of an ASI neuron, revealing very little overlap. (B) Fluorescence images of DLK-1::GFP (using *p*_*gpa-4*_::*dlk-1*::*gfp*) and immunofluorescence staining of the Golgi protein SQL-1, revealing very little overlap. Scale bar is 1 μm.
(TIF)

S5 Fig GFP::RAB-5 does not affect dye filling, cilium length or IFT in ASI neurons. (A) Merge of bright field image and fluorescence images of DiI dye filling, GFP::RAB-5 (using *p*_*gpa-4*_::*gfp*::*rab-5)* and these two together, revealing dye filling in the ASI neurons that express GFP::RAB-5. Anterior is to the left. (B) Average lengths of sensory cilia of the ASI neurons in wild type animals and in animals expressing GFP::RAB-5 in the ASI neurons (average of two strains). No statistical significant difference was observed (p>0.05). (C) Average speeds of OSM-3::mCherry (using *p*_*gpa-4*_::*osm-3*::*mCherry*) or KAP-1::mCherry (using *p*_*gpa-4*_::*kap-1*::*mCherry*) expressed specifically in the ASI neurons, together with GFP::RAB-5 (using *p*_*gpa-4*_::*gfp*::*rab-5)*. Two OSM-3::mCherry expressing strains and two KAP-1::mCherry expressing strains were analyzed. No statistical significant differences were observed between OSM-3::mCherry and KAP-1::mCherry particle speeds (p>0.05). Speeds were very similar to those reported in wild type animals (e.g. in [10, 21]). Error bars indicate SD.
(TIF)

S6 Fig GFP::RAB-5 accumulates in AWB neurons in *pmk-3* mutant animals. (A) Fluorescence images of dendrite endings and cell bodies of AWB neurons expressing GFP::RAB-5 (*p*_*str-1*_::*gfp*::*rab-5*) in wild type and *pmk-3(ok169)* animals, imaged using the spinning disk microscope. Exposure time and laser intensity were kept constant. Scale bar 1 μm. (B) Mean fluorescence intensities of dendritic endings of wild type and *pmk-3(ok169)* animals, quantified using ImageJ. 7 animals were imaged per genotype. Peak of *pmk-3(ok169)* (red *) is significantly different from that of wild type (p<0.001). Statistical analysis was performed using a t-Test. Error bars represent SEM.
(TIF)

S1 Video DLK-1::GFP motility in the dendrite of an ASI neuron. Time-lapse video (7 frames/second) of the end of the dendrite of a wild type animal expressing DLK-1::GFP in the ASI neurons, revealing punctae moving along the dendrite. Video was acquired using a spinning disk microscope with a 100x objective. Exposure time 300 ms, 150 frames. Anterior is to the right.
(AVI)

S2 Video DLK-1::GFP motility in the cell body of an ASI neuron. Time-lapse video (7 frames/second) of the cell body of a wild type animal expressing DLK-1::GFP in the ASI neurons, revealing punctae moving in the cell body. Video were acquired using a spinning disk microscope with a 100x objective. Exposure time 300 ms, 150 frames.
(AVI)
