# Supplementary figures and images for: DLK-1/p38 MAP Kinase Signaling Controls Cilium Length by Regulating RAB-5 Mediated Endocytosis in Caenorhabditis elegans
Source: PLoS Genet. 2015 Dec 11;11(12):e1005733. doi: 10.1371/journal.pgen.1005733 (PMC4686109; doi:10.1371/journal.pgen.1005733)

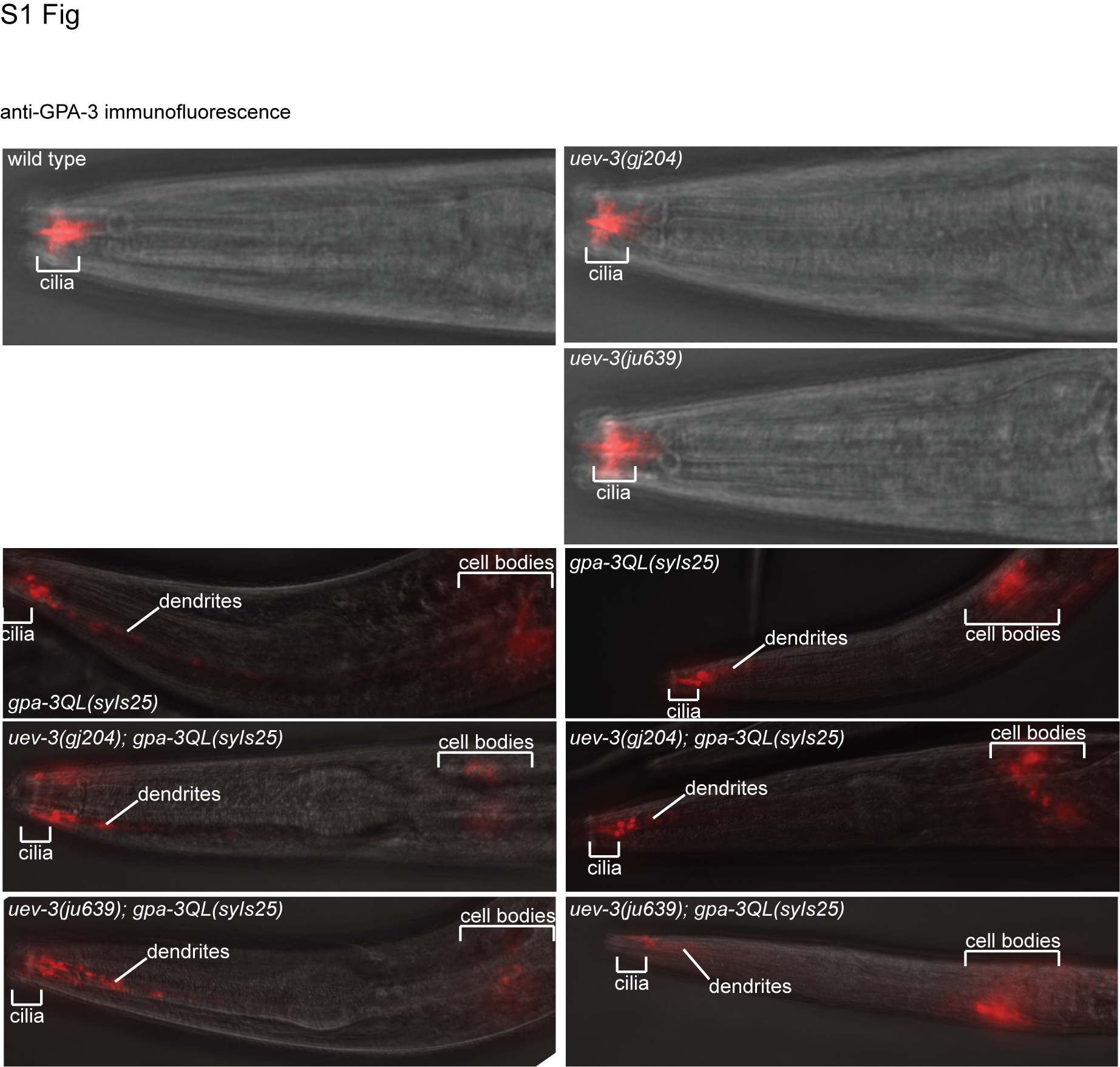

Supplement: S1 Fig — Merge of fluorescence and DIC images of wild type, uev-3(gj204) and uev-3(ju639) animals and two images of gpa-3QL(syIs25), uev-3(ju639); gpa-3QL(syIs25) and uev-3(gj204); gpa-3QL(syIs25) animals stained with anti-GPA-3 antibodies. GPA-3 localizes to the cilia in wild type, uev-3(gj204) and uev-3(ju639) animals. GPA-3 levels are strongly increased in gpa-3QL animals, resulting in anti-GPA-3 staining in dendrites and cell bodies. This anti-GPA-3 staining is not affected by mutation of uev-3. Anterior is towards the left. Exposure time was kept constant. (TIF) [file pgen.1005733.s001.tif]

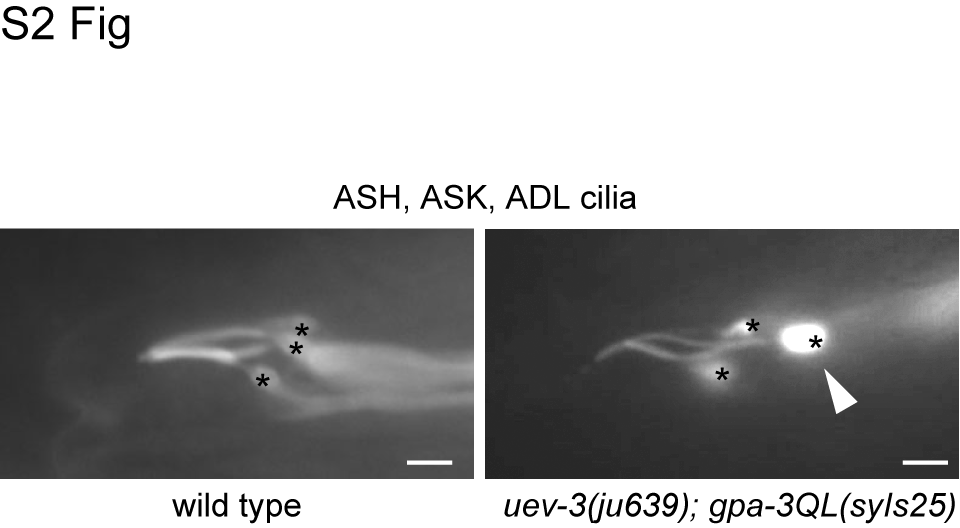

Supplement: S2 Fig — Fluorescence images of GFP in cilia of ASH, ASK and ADL neurons of wild type and uev-3(ju639); gpa-3QL(syIs25) worms using p gpa-15::gfp. In gpa-3QL animals cilia are often posteriorly displaced (Burghoorn et al, 2010). Similar displacement was observed in uev-3(ju639); gpa-3QL(syIs25) worms (arrowhead). Scale bar 2 μm. (TIF) [file pgen.1005733.s002.tif]

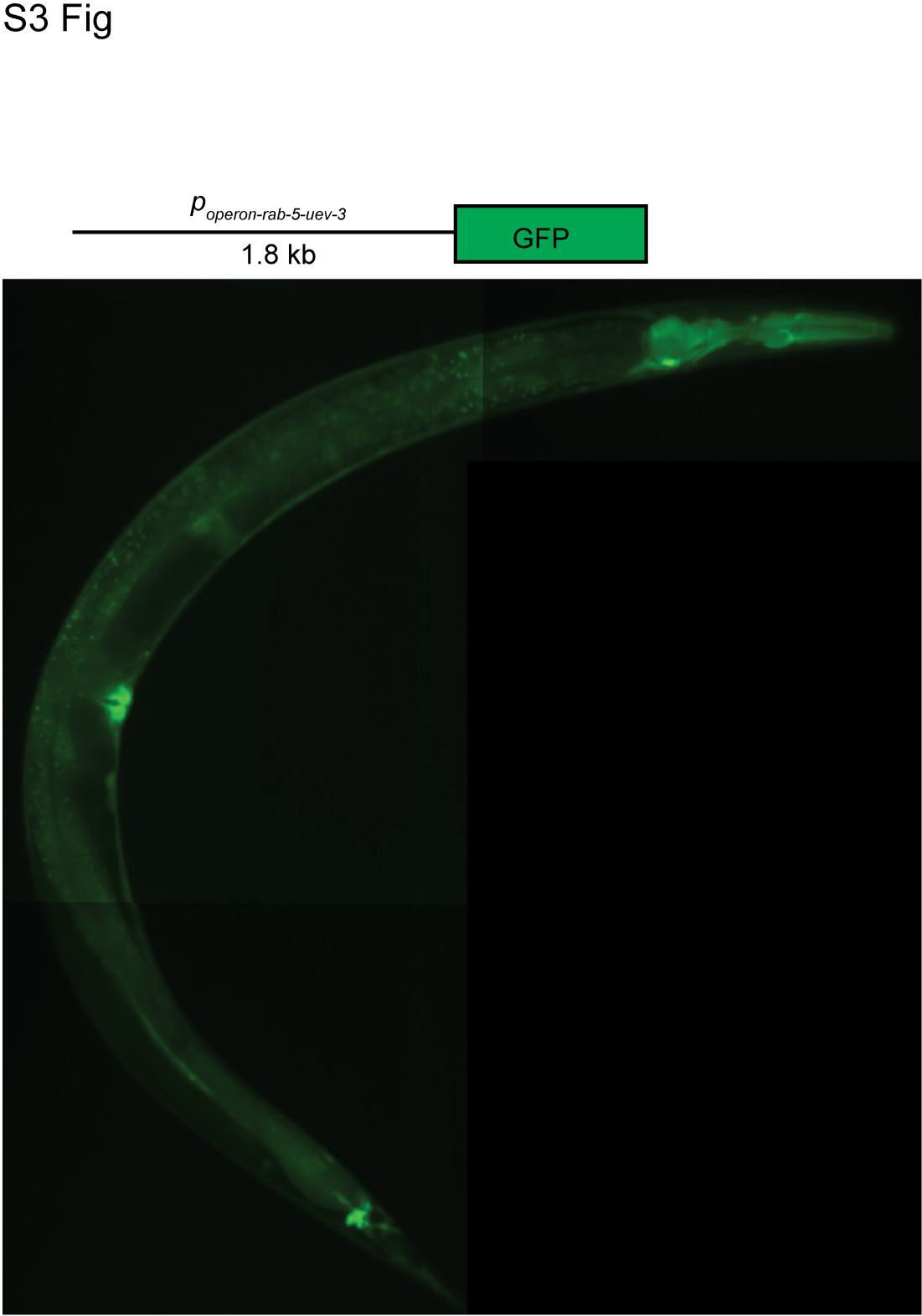

Supplement: S3 Fig — Fluorescence image of adult worm expressing UEV-3::GFP from the 1.8-kb promoter of the rab-5-uev-3 operon, schematically depicted above the image (pOF163; Trujillo et al, 2010). (TIF) [file pgen.1005733.s003.tif]

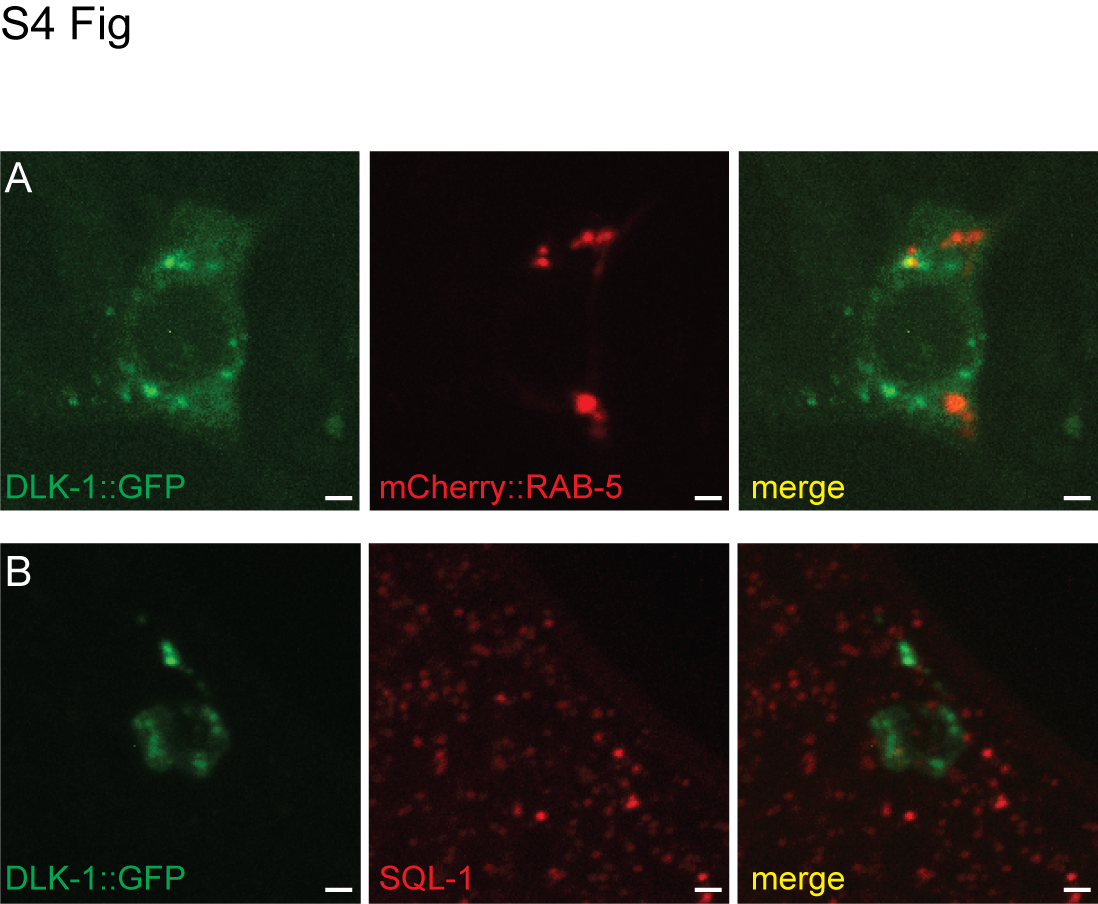

Supplement: S4 Fig — (A) Fluorescence images of DLK-1::GFP (using p gpa-4::dlk-1::gfp) and mCherry::RAB-5 (using p gpa-4::mCherry::rab-5) in the cell body of an ASI neuron, revealing very little overlap. (B) Fluorescence images of DLK-1::GFP (using p gpa-4::dlk-1::gfp) and immunofluorescence staining of the Golgi protein SQL-1, revealing very little overlap. Scale bar is 1 μm. (TIF) [file pgen.1005733.s004.tif]

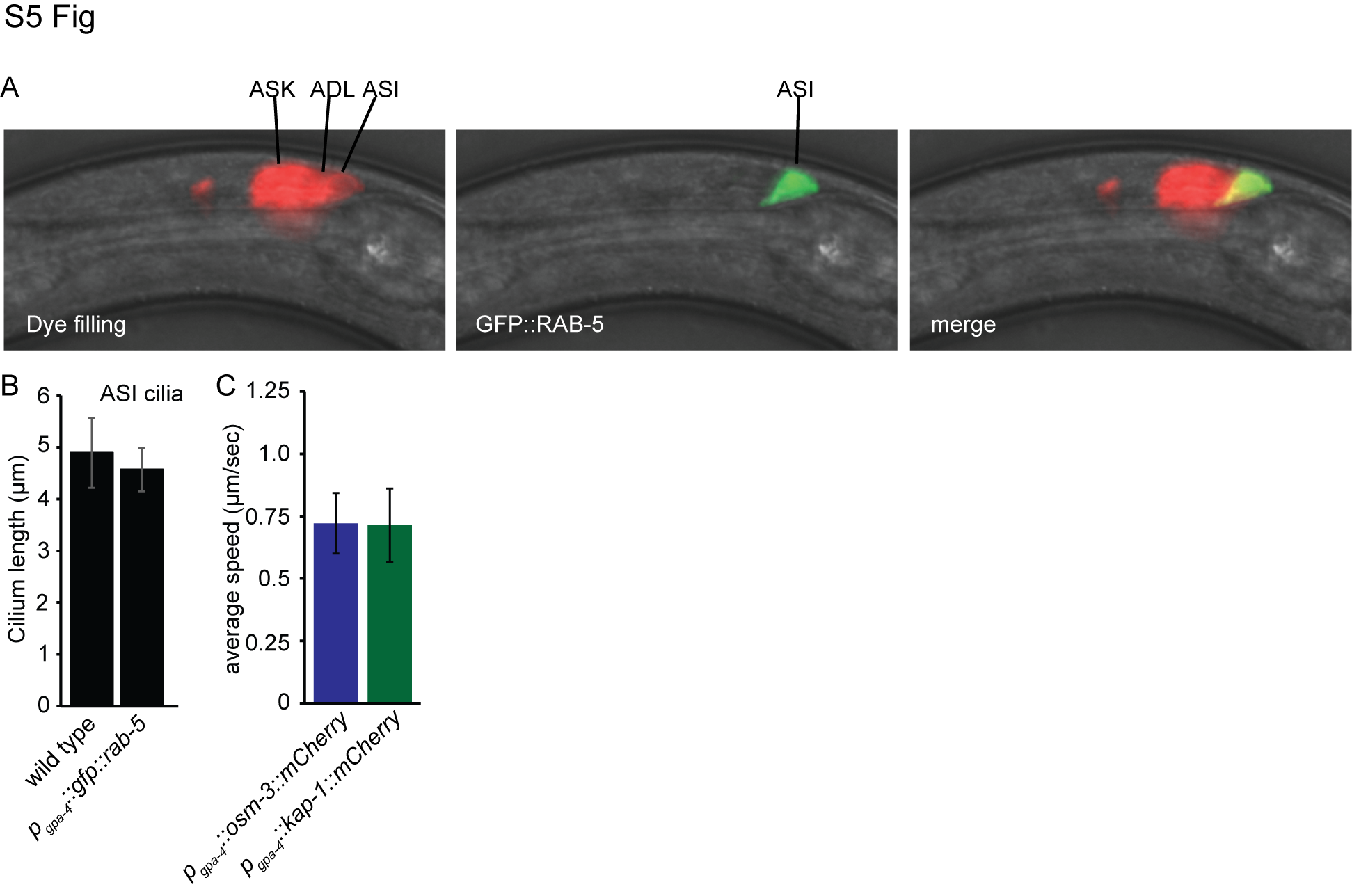

Supplement: S5 Fig — (A) Merge of bright field image and fluorescence images of DiI dye filling, GFP::RAB-5 (using p gpa-4::gfp::rab-5) and these two together, revealing dye filling in the ASI neurons that express GFP::RAB-5. Anterior is to the left. (B) Average lengths of sensory cilia of the ASI neurons in wild type animals and in animals expressing GFP::RAB-5 in the ASI neurons (average of two strains). No statistical significant difference was observed (p>0.05). (C) Average speeds of OSM-3::mCherry (using p gpa-4::osm-3::mCherry) or KAP-1::mCherry (using p gpa-4::kap-1::mCherry) expressed specifically in the ASI neurons, together with GFP::RAB-5 (using p gpa-4::gfp::rab-5). Two OSM-3::mCherry expressing strains and two KAP-1::mCherry expressing strains were analyzed. No statistical significant differences were observed between OSM-3::mCherry and KAP-1::mCherry particle speeds (p>0.05). Speeds were very similar to those reported in wild type animals (e.g. in [10, 21]). Error bars indicate SD. (TIF) [file pgen.1005733.s005.tif]

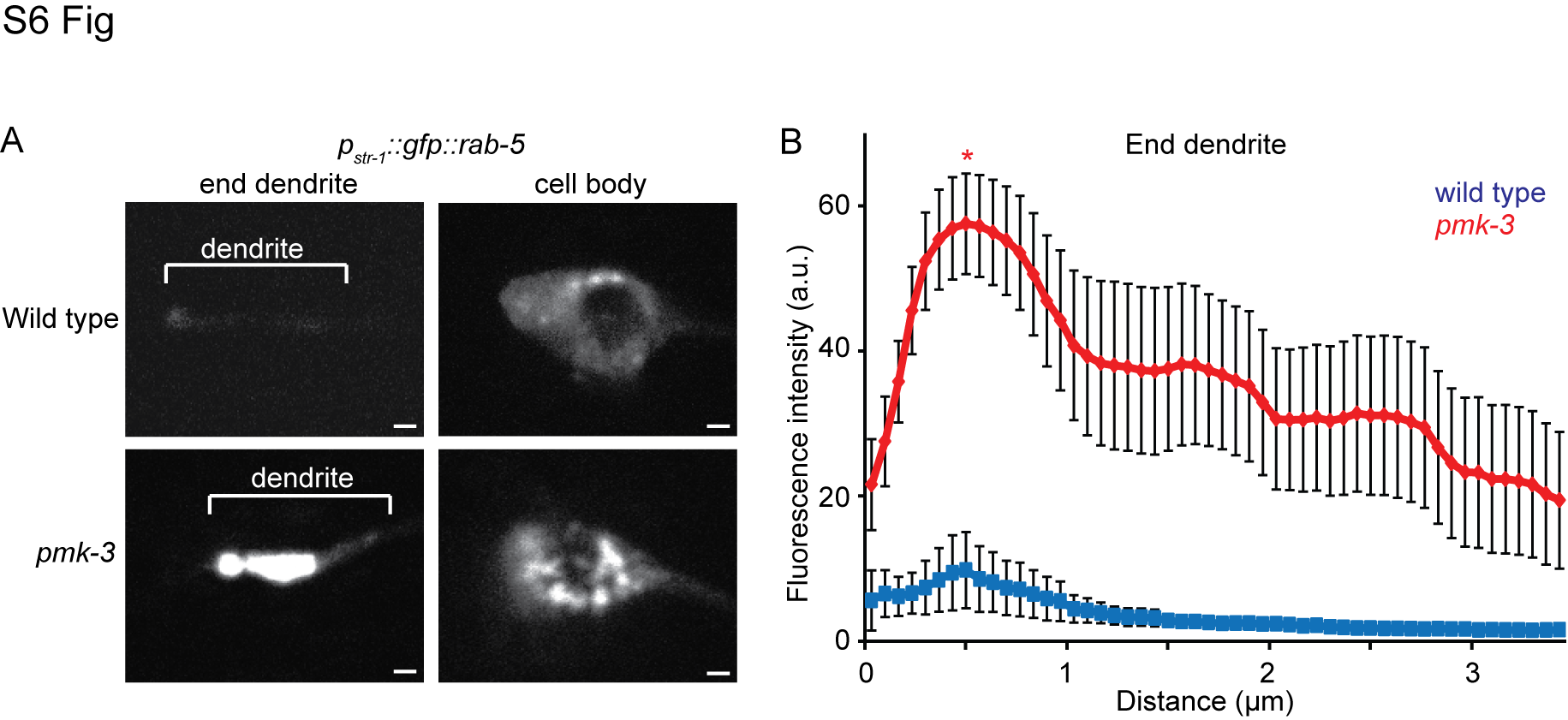

Supplement: S6 Fig — (A) Fluorescence images of dendrite endings and cell bodies of AWB neurons expressing GFP::RAB-5 (p str-1::gfp::rab-5) in wild type and pmk-3(ok169) animals, imaged using the spinning disk microscope. Exposure time and laser intensity were kept constant. Scale bar 1 μm. (B) Mean fluorescence intensities of dendritic endings of wild type and pmk-3(ok169) animals, quantified using ImageJ. 7 animals were imaged per genotype. Peak of pmk-3(ok169) (red *) is significantly different from that of wild type (p<0.001). Statistical analysis was performed using a t-Test. Error bars represent SEM. (TIF) [file pgen.1005733.s006.tif]
